# Morphofunctional Heterogeneity and Plasticity of Glioblastoma Cells Induced to Senescence by Temozolomide

**DOI:** 10.1111/acel.70477

**Published:** 2026-04-10

**Authors:** Solon Andrades da Rosa, Henrique Quaiato de Oliveira, Laura Boose de Mendonça, Nicole Borgmann de Oliveira, Mariane da Cunha Jaeger, Luiza Cherobini Pereira, Fernanda Dittrich Pinto Oliveira, Debora Santos‐Sousa, Fernanda Saez‐Calazans, Luana Lenz, Melike Lakadamyali, Guido Lenz, Eduardo Cremonese Filippi‐Chiela

**Affiliations:** ^1^ Programa de Pós‐Graduação Em Biologia Celular e Molecular, Universidade Federal do Rio Grande do Sul Porto Alegre Rio Grande do Sul Brazil; ^2^ Centro de Biotecnologia, Universidade Federal do Rio Grande do Sul Porto Alegre Rio Grande do Sul Brazil; ^3^ Departamento de Biofísica Instituto de Biociências, Universidade Federal do Rio Grande do Sul Porto Alegre Rio Grande do Sul Brazil; ^4^ Instituto Do Câncer Infantil Porto Alegre Rio Grande do Sul Brazil; ^5^ Programa de Pós‐Graduação Em Ciências Farmacêuticas, Universidade Federal de Santa Maria Santa Maria Rio Grande do Sul Brazil; ^6^ Department of Physiology Perelman School of Medicine, University of Pennsylvania Philadelphia Pennsylvania USA; ^7^ Serviço de Pesquisa Experimental, Hospital de Clínicas de Porto Alegre Porto Alegre Rio Grande do Sul Brazil; ^8^ Departamento de Ciências Morfológicas Universidade Federal do Rio Grande do Sul Porto Alegre Rio Grande do Sul Brazil

**Keywords:** autophagy, cellular senescence, heterogeneity, phenotypic plasticity, senolytics

## Abstract

Several chemotherapeutics induce cancer cells to senescence, a persistent growth‐arrest state associated with poor cancer prognosis. Relevant features in cancer cell biology, such as phenotypic plasticity and intercellular variability, are poorly understood for senescent cells (SnCs). This study examined the morphofunctional heterogeneity and dynamics of glioblastoma cells induced to senescence by Temozolomide (TMZ), focusing on pro‐survival mechanisms, including autophagy and anti‐apoptotic proteins, and phenotypic plasticity. TMZ triggered a proliferative arrest with canonical features of senescence. Two distinct morphotypes emerged with different kinetics: extension‐rich (E‐state) cells, which were predominant early on, and flattened (F‐state) cells, which accumulated over time. These states were interchangeable, mostly from E‐state to F‐state, as revealed by single‐cell tracking. F‐state cells exhibited progressive enlargement of cellular and nuclear area, beyond extended survival, suggesting a more stable senescent phenotype despite lower p16 and autophagy levels than E‐state cells. Late autophagy inhibition using hydroxychloroquine broadly sensitized both morphotypes, reducing enlarged cells. Otherwise, early autophagy inhibition with 3‐methyladenine was not cytotoxic but led to E‐state accumulation over F‐state cells, suggesting an impact on morphometric dynamics. Beyond autophagy and p16, F‐state cells also expressed lower levels of anti‐apoptotic Bcl‐2 proteins, indicating differential activation of survival pathways. Notably, the senolytics dasatinib preferentially eliminated E‐state cells. These findings highlight the plasticity and heterogeneity of TMZ‐induced senescent glioblastoma cells and emphasize the need for selective senotherapeutic strategies aiming to attenuate the pro‐tumor effects exerted by SnCs on the tumor microenvironment.

## Graphical abstract

Glioblastoma cells induced to senescence by Temozolomide are heterogeneous, with the emergence of two main states, named E‐state and F‐state, which differ in cellular morphometry, survival mechanisms, and sensitivity to autophagy modulators and senolytics. These states are dynamic, with the occurrence of morphofunctional transitions.

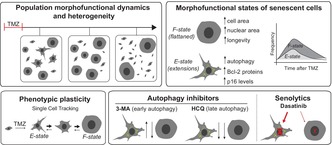

## 1 Introduction

SnCs are characterized by permanent loss of proliferative capacity triggered by telomere erosion (replicative senescence) or prematurely induced by DNA damage, oncogene overactivation, loss of tumor suppressors, and other stresses. In cancer, cellular senescence acts as an endogenous chemopreventive event, avoiding the proliferation of aged or damaged cells harboring genomic alterations (Vargas 2012). However, despite not proliferating, SnCs secrete extracellular vesicles and a myriad of soluble molecules that constitute the Senescence‐Associated Secretory Phenotype (SASP), which is enriched in growth factors, cytokines, chemokines, and matrix metalloproteinases (Childs et al. 2017; Bitencourt et al. 2024). Through that, SnCs can modulate the activity and plasticity of other cells and the adjacent extracellular matrix in several pathophysiological conditions (Calcinotto et al. 2019; Schmitt et al. 2022; Silva et al. 2024). In human cancers, including glioblastoma, higher levels of senescence are associated with poor survival (Huang et al. 2023; Liu et al. 2023; Shao et al. 2023; Wu et al. 2023; Santos‐Sousa et al. 2025). This association is relevant because, despite being resistant to replicative senescence, cancer cells are sensitive to premature senescence induced by chemotherapeutics, radiotherapy, and targeted therapies (Duy et al. 2021; Meng et al. 2021; Prasanna et al. 2021; Yasuda et al. 2021; Yamagishi et al. 2022; Salam et al. 2023).

SnCs have morphofunctional changes in size, shape, and intracellular compartments (Schmitt et al. 2022; Bitencourt et al. 2024). Furthermore, adaptive processes such as autophagy and endoplasmic reticulum stress are also altered and necessary for senescence progression and maintenance (Kanzawa et al. 2004; Natsumeda et al. 2011; Filippi‐Chiela et al. 2015; Slobodnyuk et al. 2019; Anerillas et al. 2023; Bashiri et al. 2023). SnCs also increase the expression of signaling pathways allowing apoptosis resistance (Gorgoulis et al. 2019; Behmoaras and Gil 2021; Ho and Dreesen 2021; Hanahan 2022; Yang et al. 2024). In this scenario, the overexpression of Bcl‐2 family proteins is the main effector mechanism. Therefore, Bcl‐2 anti‐apoptotic proteins are the main target for senolytics, strategies that selectively kill SnCs aiming to reduce their deleterious effects in pathophysiological conditions (Zhang et al. 2021; Beltzig, Christmann, and Kaina 2022; Rahman et al. 2022; Malayaperumal et al. 2023; Haas et al. 2025; Mayo Clinic 2026).

Although the phenotype of SnCs is well characterized, their heterogeneity and phenotypic dynamics still need to be better understood, especially regarding morphofunctional features. Senescent cells are heterogeneous across multiple dimensions, including cell type and the nature of the senescence‐inducing stimulus (Hirose et al. 2001, 2005; Günther et al. 2003; Filippi‐Chiela et al. 2013; Hernandez‐Segura et al. 2017; Aasland et al. 2019; Beltzig, Schwarzenbach, et al. 2022; Cohn et al. 2023; Liu et al. 2024; Mansfield et al. 2024). Complementarily, the expression of Bcl‐2 proteins (Troiani et al. 2022) and SASP molecules (Hernandez‐Segura et al. 2017) are also varied, even intrapopulationally. Regarding phenotypic plasticity, the transcriptome of SnCs varies over time, culminating in the emergence of subpopulations with divergent transcriptomes, mostly observed in aging studies (Kundu et al. 2020; Buj et al. 2021; Roger et al. 2021; Palmer et al. 2022; Bitencourt et al. 2024). However, most evidence about the heterogeneity and phenotypic dynamics of SnCs are based on transcriptomic analyses or populational data (Sharpless and Sherr 2015; Casella et al. 2019; Saul and Kosinsky 2021). Structural and functional evidence of these features in individual SnCs is still lacking.

In this work, using a glioblastoma cell model expressing nuclear and cytoplasmic fluorescent reporters, we comprehensively characterized heterogeneity and phenotypic dynamics of cells induced to senescence by Temozolomide (TMZ), the first‐choice chemotherapy in glioblastoma and classic inducer of senescence. We explored the morphofunctional states of individual SnCs as well as survival mechanisms and the sensitivity of SnCs living in distinct states to autophagy modulators and senolytics.

## 2 Results

### 2.1 TMZ Induces Transient Enrichment of SnCs Followed by Population Regrowth of Glioblastoma Cells

**FIGURE 1:**
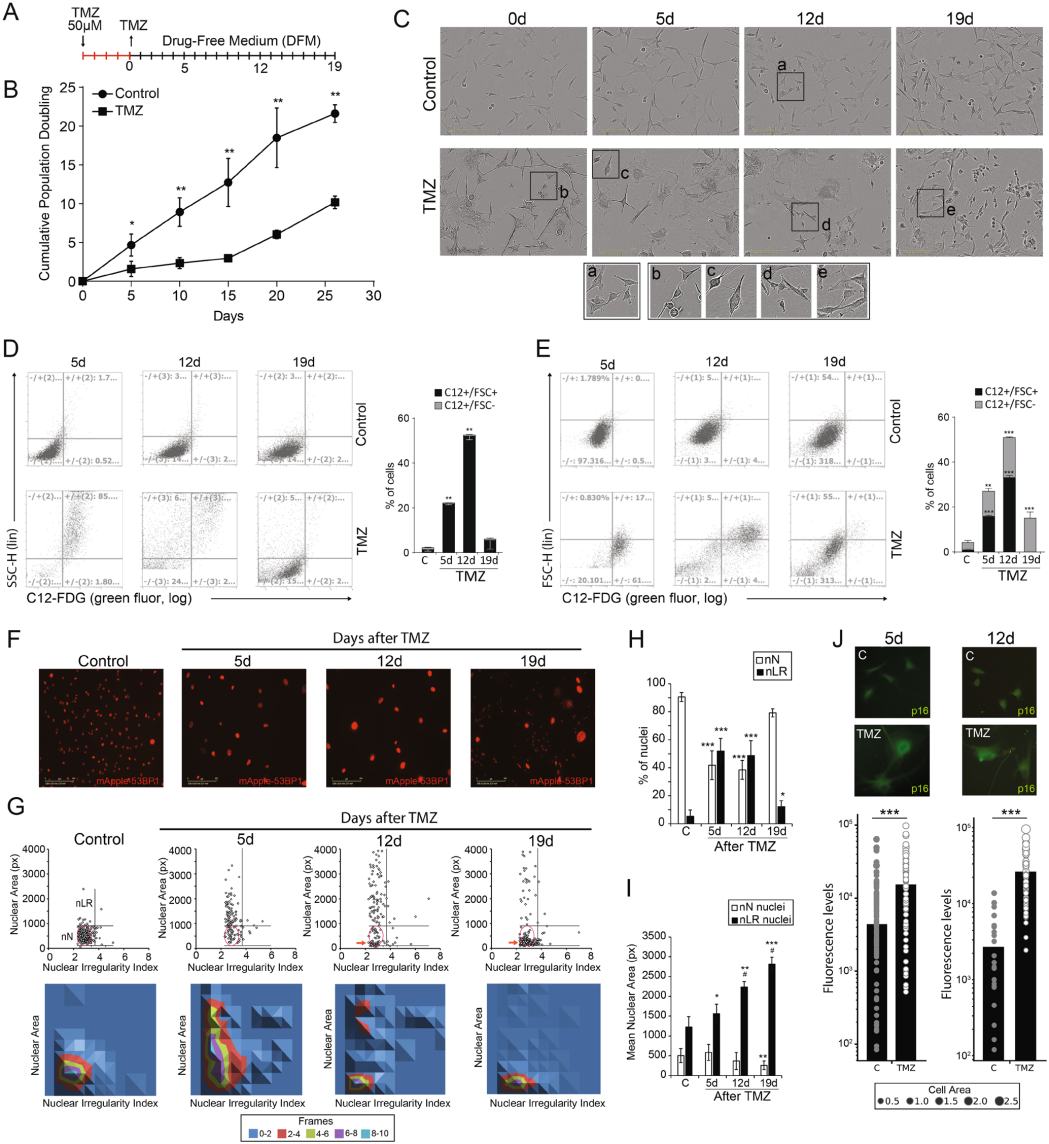
Temozolomide induces enrichment of SnCs followed by regrowth of U‐87 glioblastoma cells. (A) Experimental design. Cells were treated with TMZ 50 μM for 5 days, followed by growth in Drug‐Free Medium for 19 days. Most analyses were performed 5d, 7d, and 19d after the treatment. (B) Cumulative Population Doubling (CPD), obtained through U87 wt cells. (C) Representative bright field images of control and TMZ‐treated U87 wt cells. (D and E) Flow cytometry for (D) C12‐FDG versus FSC (cell size) or (E) C12‐FDG versus SSC (cell granularity) for control and TMZ‐treated cells over time. Left—plots with quadrants defining the four subpopulations; right—bar plots showing the percentage of cells in each quadrant. (F) Representative images of nuclear morphology observed for red fluorescence (mApple53BP1) obtained with U‐87 GFP‐LC3 mApple‐53BP1 cells (U‐87LC3‐53BP1). (G) Nuclear Morphometric Analysis (NMA). Top—Area versus Nuclear Irregularity Index (NII) scatterplot. nN, Normal; nLR, Large Regular. Bottom—NMA density plot. See also Figure S1A, which depicts the NMA graph. (H) Percentage of Normal and Large Regular nuclei. (J) Mean nuclear area of Normal and Large Regular nuclei. (I) p16 levels after 5d and 12d were measured in individual U87 wt cells by immunocytochemistry. Top—representative images for control and TMZ.

U‐87 wt cells were treated with TMZ 50 μM for 5 days, followed by 21 days of recovery in Drug‐Free Medium (DFM) to simulate the clinical schedule (Figure 1A). After TMZ, the cell population transiently stopped growing (Figure 1B), concomitant to the enrichment of morphological changes resembling cellular senescence, like cell enlargement, cytoplasm flattening, and membrane extensions (Figure 1C). A subset of proliferative cells, with morphology similar to control cells but with reduced size, emerged from Day 12 onwards (Figure 1B,C).

We then explored the dynamics and heterogeneity of typical cellular senescence markers. U87 wt cells were treated in the same clinical schedule and then we observed an early increase in SA‐β‐gal‐positive cells with high intracellular granularity (i.e., SSC) 5 days after TMZ (Figure 1D), followed by cellular enlargement (i.e., FSC) of SA‐β‐gal‐positive cells on Day 10 (Figure 1E). TMZ‐treated cells also increased nuclear area, another hallmark of senescence (Filippi‐Chiela et al. 2012) (Figure 1F). Through the Nuclear Morphometric Analysis (NMA), we classified individual nuclei based on Nuclear Area and Nuclear Irregularity Index (NII) using U‐87 GFP‐LC3 mApple‐53BP1 cells (U‐87LC3‐53BP1) (Figure 1G; Figure S1A). The NMA uses nuclear area and shape measurements to classify each nucleus as normal (nN), large regular (nLR), large irregular (nLI), irregular (nI), small regular (nSR), or small irregular (nSI). Large (nLR and nLI) nuclei are associated with cellular senescence; irregular nuclei (nI) are associated with irregularities across multiple scenarios, such as mitotic catastrophe or mitotic slippage, and small nuclei are associated with apoptosis (nSR) or mitosis (nSI); irregular nuclei (LI and I) are associated with irregularities across multiple scenarios, such as mitotic catastrophe or mitotic slippage; and small nuclei are associated with apoptosis (nSR) or mitosis (nSI) (Filippi‐Chiela et al. 2012). The separation of these categories is based on the distribution of control nuclei and the definition of the normal ellipse, where 90% to 95% of the control nuclei are positioned. The horizontal thresholds that separate large and small nuclei are defined by the upper and lower limits of the normal ellipse, respectively. The vertical thresholds that separate populations based on irregularity are determined by the density patterns of nuclear subpopulations. The rationale for determining thresholds is based on flow cytometry gating strategies. The user should enrich the graph with as much data as possible to better define the cell populations and, consequently, the position of the thresholds. The user places thresholds at the limits of each enriched subpopulation in the graph. The criteria and strategies for defining appropriate thresholds, as well as examples of incorrectly set thresholds, are presented in detail in the original NMA article (Filippi‐Chiela et al. 2012). Notably, the NMA data analysis spreadsheet, as well as CellMorph, applies the same threshold values to all analyzed conditions (control and treatments), in order to standardize the categorization of nuclei and cells. We observed a transient increase in the percentage of Large Regular nuclei (nLR) after TMZ, with concomitant reduction of normal Nuclei (nN) (Figure 1G,H). Despite the percentage of nLR being similar after 5d and 12d (Figure 1H), the mean area of nLR increased over time, suggesting phenotypic progression towards senescence (Figure 1I). Finally, a population of nN notably smaller than N nuclei from control condition, emerged on Day 12 after TMZ (Figure 1G, red arrow; Figure 1I, white bars) corroborating data from cell morphology (Figure 1C). Noteworthy, even after 19 days we still observed nLR (Figure 1G), reaching the largest average area (Figure 1I), suggesting a long‐lasting survival of SnCs. Finally, TMZ‐treated U87 wt cells also increased p16 levels (Figure 1J). In conclusion, exposure to TMZ resulted in a transient growth arrest of glioblastoma cells followed by regrowth. The proliferative arrest coincided with the transient increase of senescence markers with variable kinetics and intercellular heterogeneity.

### 2.2 TMZ‐Induced Enlarged Cells Are Heterogeneous and Phenotypically Dynamic

**FIGURE 2:**
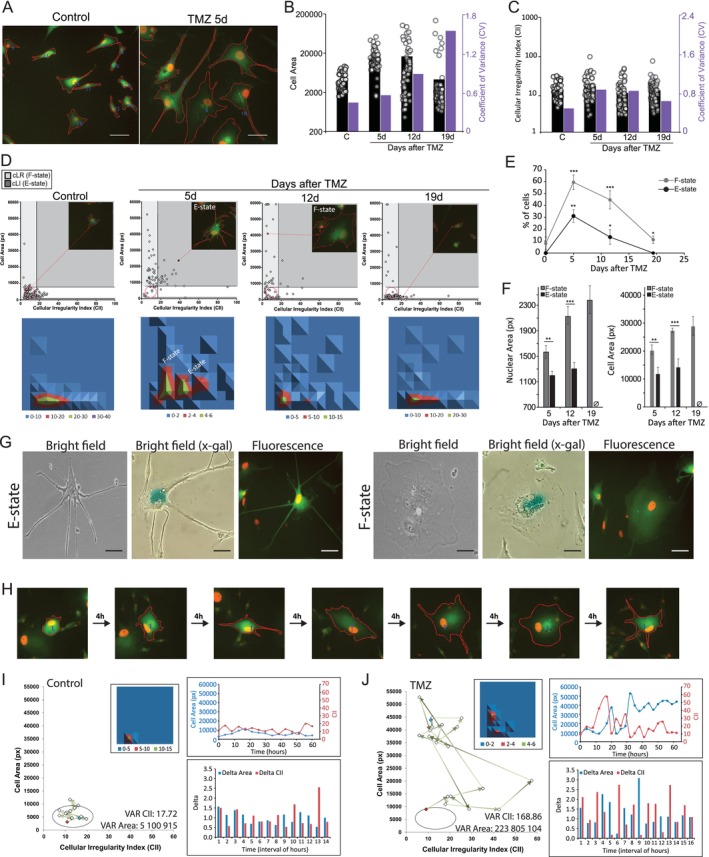
TMZ induces the transient enrichment of 2 distinct and dynamic morphological states considering enlarged cells. (A) Representative images of segmented U‐87LC3‐53BP1cells. The red line represents the cell outline. (B) Area and (C) Cellular Irregularity Index (CII) of individual cells from control and TMZ conditions. Purple bars indicate the coefficient of variance (CV) for each condition. (D) CellMorph analysis. Top—Cell Area versus CII scatterplot. Light gray—Large Regular cells (cLR), which represent F‐state cells; dark gray—Large Irregular cells (cLI), which represent E‐state cells. In each graph, a representative cell is indicated as a red diamond marker and shown as an insert on the top right. Bottom—CellMorph density plot. E‐state and F‐state subpopulations are indicated. See also Figure S1B, which depicts the CellMorph graph. (E) Percentage of F‐state and E‐state cells over time after TMZ treatment. (F) Differential Nuclear Area and Cell Area of E‐state and F‐state cells over time. The Ø icon indicates the absence of E‐state cells. (G) Representative brightfield, X‐gal staining, and fluorescence images for F‐state and E‐state cells. (H) Single‐cell tracking example. The cell contour resulting from the segmentation is shown as a red line. (I and J) TrackingCellMorph to individual cells from (I) Control or (J) TMZ condition over time. Left graphs—CellMorph (CII versus Cell Area scatterplot). Red and blue markers represent the starting and ending points of the tracking, respectively. Arrows connecting the dots indicate the phenotypic trajectories of the cells. The insert on the top right shows the density plot demonstrating phenotypic regions most occupied by cells. The Variance (VAR) of Cell Area and CII is also shown. Right graphs—top: Cell Area and CII oscillation over time; bottom: Delta values for Cell Area and CII over time.

SnCs are transcriptionally heterogeneous (Hernandez‐Segura et al. 2017; Troiani et al. 2022). This phenotypic variability was also observed in morphometric and subcellular features in our model. Considering that the morphofunctional heterogeneity and plasticity of SnCs have been neglected, we then advanced to explore those features in TMZ‐treated U‐87 GFP‐LC3 mApple‐53BP1 (U‐87^LC3‐53BP1^) cells. Initially, we performed Cellular Morphometric Analysis (CellMorph), which classifies individual cells based on cell area and regularity (Figure S1B). The rationale for defining thresholds and separating quadrants in CellMorph follows the same rationale as in NMA. As in flow cytometry analysis using a scatter plot, after defining the normal ellipse that encompasses 90 to 95% of the cells in the control condition, the vertical thresholds (including the one separating the LR and LI quadrants) are placed at the boundary separating enriched subpopulations in the graph. Details on this placement, including correct and incorrect examples of separation, are demonstrated in the original article (Quaiato De Oliveira et al. 2025). Representative images of segmented cells are shown on Figure 2A. We confirmed the increase in cell area on Day 5 and 12 after treatment, with increased variability in cell area (Figure 2B) and shape (Figure 2C) compared to control. Corroborating data from NMA (Figure 1H), small cells emerged on Day 12 after TMZ, becoming the dominant phenotype after 19d (Figure 2B).

Enlarged cells grouped into two main morphostates on Day 5 after TMZ (Figure 2D). Enlarged cells with high CII had prominent extensions (named E‐state cells) and occupied the Large Irregular (cLI) quadrant (Figure 2D). Enlarged cells with low CII, in turn, were characterized by a flattened cytoplasm (named F‐state cells) and occupied the Large Regular (cLR) (Figure 2D). This predominance of two states peaked on Day 5 after TMZ treatment (Figure 2E), corroborating data from NMA. Corroborating this data, we treated another glioblastoma lineage, A172 (Figure S2A) which exhibited a very similar distribution to U87 cells (Figure S2B,C). Curiously, a primary glioblastoma lineage treated with a similar protocol (Figure S2D,E) also showed either the E and F‐state distributions (Figure S2F,G). After 19 days, enlarged cells became rare as proliferative cells emerged, and only F‐state cells were observed, suggesting a longer‐lived phenotype. Finally, F‐state cells had higher nuclear area and cellular area compared to E‐state cells, and F‐state cells that survived up to 19d showed progressive cellular and nuclear enlargement (Figure 2F). Notably, both E‐state and F‐state cells stained positively for SA‐β‐gal and had enlarged nuclei (Figure 2G).

The ability of cancer cells to vary phenotypes and switch between different states is a hallmark of cancer (Hanahan 2022). This phenotypic plasticity of SnCs, however, is unknown. Thus, we assessed the morphofunctional dynamics of individual F‐state and E‐state cells (Figure 2H). Untreated cells subtly varied in size and shape over time, remaining confined to the normal (cN) region of CellMorph (Figure 2I and Figure S3A). Conversely, TMZ‐treated cells greatly varied Area and CII over time, transitioning between E‐state and F‐state (Figure 2J and Figure S3B). Starting from a normal phenotype (cN), most cells passed through E‐state before reaching F‐state, which may be long‐lasting (Figure 2J; Figure S3B: TMZ#1 to TMZ#6). However, some cells switched from F‐state back to E‐state (e.g., TMZ#7 and TMZ#8), suggesting great plasticity for SnCs. Interestingly, none of the enlarged tracked cells proliferated during the tracking experiments, from Day 5 to Day 10 after treatment.

In conclusion, TMZ‐induced SnCs showed significant morphological heterogeneity and phenotypic plasticity. Two main states predominated, with F‐state cells showing a more stable phenotype, exhibiting progressive nuclear and cellular enlargement.

### 2.3 Differential Association Between Senescent Morphofunctional States and Cell Cycle Status and Proliferativeness of Enlarged Cells

Despite extensive characterization of senescent cells, the molecular pattern associated with the different phases of the cell cycle by senescent cells remains incompletely characterized, particularly among senescent cells exhibiting different morphofunctional states. The gene signature (RNA) expression profile of different phases of the cell cycle is heterogeneous among cells with transcriptomic characteristics of senescence (Mao et al. 2012; Tao et al. 2024; Ahn et al. 2025), which is associated with differences in the levels of senescence markers (Neri et al. 2025). We investigated the relationship between the molecular pattern of gene expression associated with the cell cycle and the morphofunctional states of senescent cells. For this, we used U87 cells stably expressing the FUCCI reporter (Figure S4A), a widely used tool for assessing cell cycle status in cycling cells that measures the activity of the ubiquitin‐proteasome system targeting CDT1 and Geminin. G1‐FUCCI cells exhibit red nuclei, cells entering S phase exhibit yellow nuclei, and G2/M‐FUCCI cells exhibit green enlarged nuclei (Sakaue‐Sawano et al. 2008; Marcus et al. 2015). Integrating NMA with FUCCI fluorescence (fucciNMA) produced a cell cycle distribution (Figure S5B) similar to the distribution obtained by flow cytometry (Figure S4B). Furthermore, control nuclei in the S and G2/M phases had a larger area than nuclei in the G1 phase (Figure S4C).

As expected, fucciNMA confirmed that TMZ increased G2/M‐FUCCI cells (Figure S4D). We then performed a multiphenotypic analysis integrating CellMorph and FUCCI at the single‐cell level. CellMorph graph confirmed the presence of E‐state and F‐state cells after TMZ (Figure S5C). To allow integrated visualization of FUCCI and morphofunctional states, we plotted G1‐FUCCI and G2/M‐FUCCI cells on separate graphs and examined the profiles of distinct morphological subpopulations (Figure S5D). Notably, considering F‐state cells (i.e., cLR quadrant), we observed a predominance of G1‐FUCCI cells (Figure S5E), while for E‐state cells (i.e., cLI quadrant), there was a slight predominance of cells in the G2/M‐FUCCI cells. Furthermore, G1‐FUCCI cells were larger compared to G2/M‐FUCCI cells (Figure S5F,G). Consistent with this, we observed subpopulations of larger cells in G1‐FUCCI cells compared to G2/M‐FUCCI cells (Figure S5C: density plots). Overall, these findings reveal a structured association between cell cycle status and senescent morphofunctional states, in which most G2/M‐FUCCI cells are in the E‐state, while G1‐FUCCI cells are predominantly F‐state cells, exhibiting larger cell size than G2/M‐FUCCI cells.

We complemented FUCCI data in A172 and U87 cells with immunocytochemistry for Histone H3 phosphorylated at Serine 10—pH3(Ser10)—, a molecular marker that triggers chromosome condensation at the end of the G2 phase, peaking at the start of mitosis (Hendzel et al. 1997). Morphologically, mitotic cells acquire a typical rounded morphology, which can be assessed using CellMorph (Quaiato De Oliveira et al. 2025). Several mitotic cells were observed in the control condition of both A172 and U87 cells, while no enlarged cell showed positive staining for p‐H3(Ser10) (Figure S6A,B). We then performed cell segmentation to objectively assess the relationship between cell morphometry and positive pH3 (Ser10) staining. No cells located in the LR (F‐state) or LI (E‐state) quadrants showed positive staining for pH3 (Figure S6C,D), This result suggests that there is no signaling initiation for mitosis in cells living in the E and F states.

Finally, we also performed a CFSE dilution assay to directly and objectively measure the relationship between proliferative and morphofunctional states. CFSE is a supravital dye that is incorporated by cells, being diluted for daughter cells with each cell division (Brenchley et al. 2003; “CFSE dilution to study human T and NK cell proliferation in vitro,” 2020). U87 and A172 cells from the control and TMZ conditions were incubated with CFSE 5 days after TMZ exposure. After 2 days of incubation, images were acquired, followed by cellular segmentation to perform a CellMorph analysis combined with CFSE fluorescence. In both lineages, cells treated with TMZ showed higher CFSE levels than the control (Figure S7A,B), corroborating data from previous assays. It is worth noting that small cells in the TMZ condition showed lower fluorescence levels, similar to control cells, suggesting proliferative activity. We then plotted CellMorph to categorize individual cells into Normal (N), F‐state, and E‐state (Figure S7C,E). From this separation, we assessed the differential levels of CFSE in the cells treated with TMZ. Data from CFSE‐CellMorph confirmed that enlarged cells have higher levels of CFSE green fluorescence compared to normal cells both in A172 (Figure S7D) and in U87 cells (Figure S7F). Notably, E‐state and F‐state cells did not differ for CFSE levels. Together, evidence from the CFSE dilution assay confirms previous evidence suggesting that not all cells treated with TMZ acquire a senescent phenotype, and that cells with a normal phenotype may be associated with a resumption of cell population proliferation, as observed in CPD.

### 2.4 Autophagy Is Heterogeneous and Transiently Increased in E‐State Cells Progressing Into the Senescent Phenotype

**FIGURE 3:**
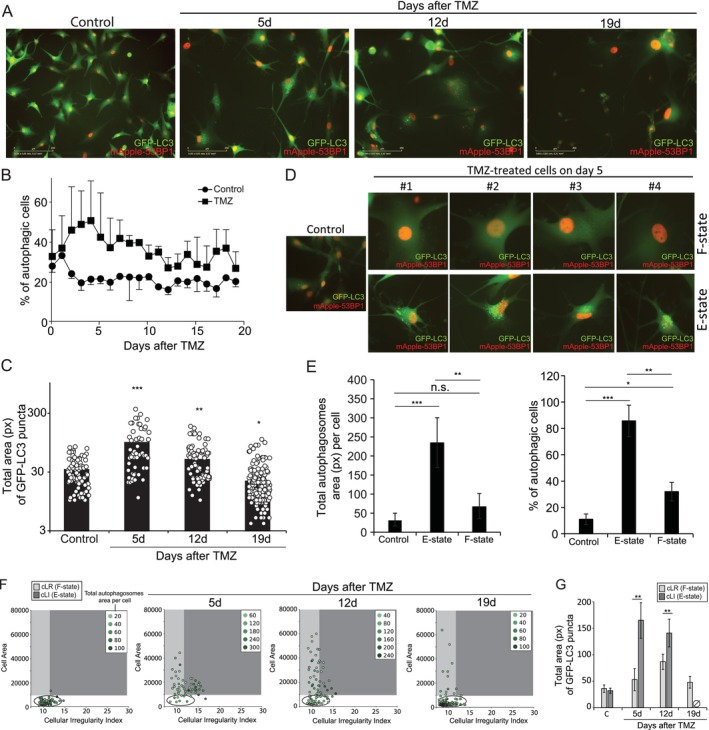
Autophagy levels are increased during the transition to increased cell size. (A) Representative images of U‐87LC3‐53BP1 showing autophagosomes as cytosolic green dots. (B) Quantification of the percentage of autophagic cells over time. Cells with at least 5 green dots were considered autophagic, in agreement with the guidelines for autophagy studies (Klionsky et al. 2021). (C) Total area of autophagosomes (i.e., GFP‐LC3 cytosolic green dots), measured in individual cells over time. To that, we used the threshold tool of FIJI to specifically quantify GFP‐LC3 cytosolic puncta. (D) Differential autophagy in F‐state and E‐state cells. Four examples of each cell state are shown. (E) *Left*—Total LC3‐GFP area per cell in E‐state and F‐state cells; *right*‐ Percentage of autophagic cells in E‐state and F‐state cells. (F) CellMorph scatterplot. The intensity of marker color represents the total area of autophagosomes in individual cells. cLR, Large Regular cells (i.e., F‐state cells); cLI, Large Irregular cells (i.e., E‐state cells); see also Figure S1B. (G) Autophagosomes area per cell in cells from cLR and cLI. The Ø icon indicates the absence of E‐state cells on Day 19 after treatment.

Autophagy allows cellular adaptation to stress (Liu et al. 2023), favoring senescence to the detriment of apoptosis (Filippi‐Chiela et al. 2015; Slobodnyuk et al. 2019). Cells that survived TMZ showed an increase in cytosolic GFP‐LC3 dots (i.e., autophagosomes), with great intercellular heterogeneity (Figure 3A). Populationally, autophagic cells reached a peak on Day 4 after TMZ treatment, followed by an oscillatory dynamic tending towards reduction (Figure 3B). Individual cells had variable autophagosome area, mainly on Day 5 after TMZ (Figure 3C), reinforcing the heterogeneous response of glioblastoma cells to TMZ.

The broad heterogeneity of SnCs and autophagy led us to investigate the differential phenotype and role of autophagy in cells living in E‐ and F‐states. Most cells in the E‐state showed numerous autophagosomes, in contrast to F‐state cells (Figure 3D). Both the percentage of autophagic cells and the area of autophagosomes in individual cells were higher for E‐state compared to F‐state cells (Figure 3E). Therefore, we next assessed the dynamics of autophagy levels along phenotypic progression to senescence. After TMZ, cells with an intermediate increase in cell area showed high levels of autophagy (Figure S8: cells above the black dashed line), whereas enlarged cells showed lower levels of autophagy, even compared to control cells (Figure S8: red square). To deeply characterize autophagy in E‐ and F‐states, we integrated cell size, shape, and autophagy levels in individual cells. We observed higher levels of autophagy in cells progressing from normal (cN) to enlarged phenotypes (cLR and cLI), at much higher levels in E‐state cells (i.e., cLI quadrant cells) compared to F‐state cells, especially those with greatly increased size (Figure 3F,G; Figure S8). This data suggests that autophagy may have a role in phenotypic progression and adaptation, but is greatly reduced in long‐lived SnCs.

### 2.5 Autophagy Inhibitors Have Differential Effects in TMZ‐Treated Glioblastoma Cells

**FIGURE 4:**
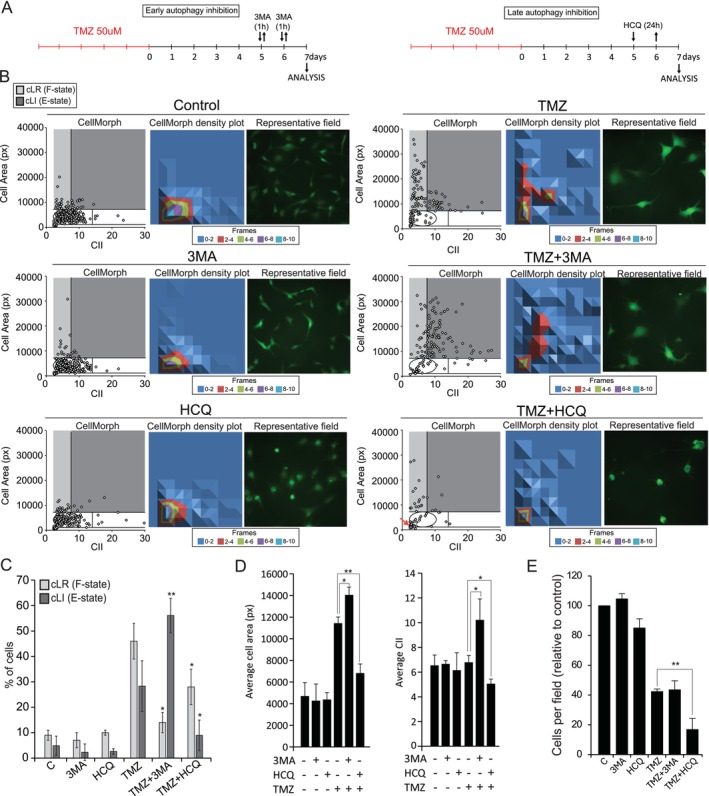
Autophagy inhibitors have differential effects in TMZ‐treated glioblastoma cells. (A) Experimental designs. Cells were treated with TMZ 50 μM for 5 days, followed by growth in Drug‐Free Medium. Left—early autophagy inhibition. TMZ‐treated cells were treated with 3‐methyladenin (3MA) for 1 h on Day 5 and Day 6 after treatment. Right—late autophagy inhibition. TMZ‐treated cells were treated with Hydroxychloroquine (HCQ) 40 μM for 24 h on Day 5 after treatment. (B) To each condition are shown: Left—CellMorph graphs are shown. Light gray—Large Regular cells, cLR (i.e., F‐state cells); dark gray—Large Irregular cells, cLI (i.e., E‐state cells). Red arrow in TMZ + HCQ indicates small regular cells. Middle—CellMorph density plot. Right—representative image. (C) Percentage of cLR and cLI cells to each condition on Day 7. (D) Average Cell Area and NII to all conditions. (E) Cell number. Data are given as the number of cells per field in relation to control (considered 100%).

Higher levels of autophagy may contribute to senescence over apoptosis after DNA damage (Filippi‐Chiela et al. 2015). Considering the transitory increase of autophagy and the varying levels of autophagy in F‐ or E‐state cells, we assessed the differential impact of early (3‐methyladenin, 3MA) or late (hydroxychloroquine, HCQ) autophagy inhibition in SnCs living in different states on Day 7 after TMZ treatment (Figure 4A). Firstly, we confirmed the emergence of subpopulations of enlarged cells on Day 7 through CellMorph (Figure 4B), corroborating data from Day 5 (Figure 2D). 3MA enriched E‐state in TMZ‐treated cells, concomitant with the reduction of cLR (Figure 4B,C). HCQ, in contrast, reduced both cLR and cLI (Figure 4B,C). HCQ increased the number of round and detached cells (Figure 4B: representative images), leading to the emergence of Small Regular (cSR) cells, likely to represent apoptotic cells. Compared to TMZ, 3‐MA promoted an average increase in cell area and, mainly, in CII. In contrast, adding HCQ to TMZ‐treated cells promoted a reduction in cell area and irregularity (Figure 4D), reducing cell number compared to TMZ alone (Figure 4E). Interestingly, both HCQ and 3‐MA, when added to control proliferative cells had no effect over shape descriptors and cell number revealing a very senescent specific effect. (Figure 4B,C). In summary, early autophagy inhibition altered the phenotypic state of enlarged cells towards cellular irregularity. In contrast, late autophagy inhibition sensitized TMZ‐treated cells, reducing all cellular senescence morphostates.

### 2.6 F‐State and E‐State SnCs Have Variable Levels of Bcl‐2 Proteins and Are Differentially Sensitive to Senolytics

**FIGURE 5:**
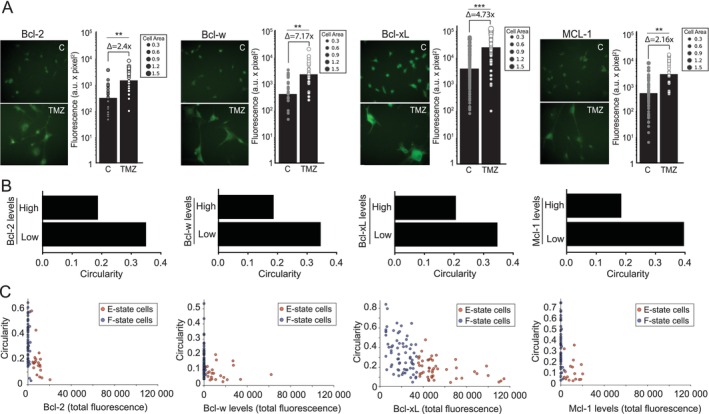
Enlarged cells living in F‐state or E‐state differentially increase levels of anti‐apoptotic Bcl‐2 proteins. (A) Immunocytochemistry for Bcl‐2, Bcl‐w, Bcl‐xL, and Mcl‐1 in U‐87 wt cells after 10d TMZ treatment. Representative images of each protein are shown on the left. Graphs show levels of each protein in individual cells and the mean levels in Control and TMZ conditions. Delta values comparing control and TMZ are also shown. (B) Levels of Bcl‐2 proteins according to the circularity. The cells were separated according to the levels of Bcl‐2 into low and high, and then circularity was calculated. (C) Scatterplots for levels of Bcl‐2 proteins and circularity. Blue and red markers represent cells with low or high levels of Bcl‐2 proteins.

Together with autophagy, the increase of Bcl‐2 anti‐apoptotic proteins is also involved in the survival of SnCs (Zhu et al. 2015; Troiani et al. 2022). We then investigated differential levels of Bcl‐2 proteins in SnC morphostates. TMZ‐treated cells increased Bcl‐2, Bcl‐w, Mcl‐1 and, mostly, Bcl‐xL (Figure 5A). F‐state cells (i.e., cells with high circularity) had lower levels of Bcl‐2 proteins than E‐state cells (Figure 5B,C). We also measured the differential levels of p16, which is involved not only in senescence establishment but also in apoptosis resistance (Li et al. 2015; Safwan‐Zaiter et al. 2022). E‐state cells consistently exhibited higher levels of p16 than F‐state cells (Figure S9).

We also measured CDKN2A promoter activation levels using a fluorescent reporter model (Figure S10A). Briefly, a CDKN2A promoter construct controlling EGFP expression, containing nuclear localization sequences, was inserted into A172 glioblastoma cells by lentiviral transduction (Figure S10A,B). This allows for the objective assessment of promoter activity levels based on fluorescence levels, using both microscopy and flow cytometry. We observed an increase in GFP levels in cells treated with TMZ compared to the control, both by microscopy (Figure S10B) and by flow cytometry (Figure S10C). In this model, we also performed immunostaining for endogenous p16 levels, confirming increased protein levels in cells treated with TMZ (Figure S10D).

Using this reporter, we also associated fluorescence levels with cell morphometry in individual cells (Figure S10E), generating an integrated multiphenotypic analysis. In this scenario, cells in E‐state showed higher levels of CDKN2A promoter activity than cells in F‐state (Figure S10E,F). Corroborating these results, we observed a positive correlation between the Cell Irregularity Index (which is higher in E‐state cells) and promoter activity levels (Figure S10G: top), while the correlation between promoter activity and cell area (which is lower in E‐state cells) was negative (Figure S10G: bottom). Finally, we leverage the model's features to provide evidence on CDKN2A promoter activity across cell cycle phases. As expected, TMZ induced a G2/M phase arrest, as shown by Propidium Iodide levels (Figure S10H,I). Notably, G2/M phase cells and polyploid cells showed progressively higher levels of promoter activity, suggesting a relationship between promoter activation and ploidy (Figure S10J).

**FIGURE 6:**
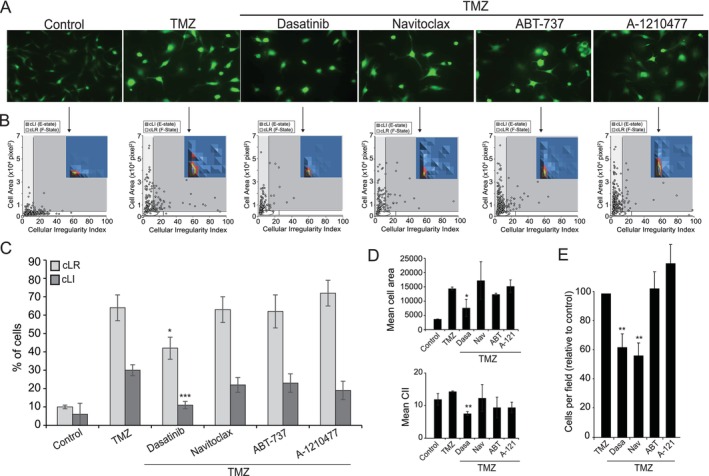
Senolytics have differential effects in TMZ‐treated glioblastoma cells. Cells were treated with senolytics on Day 10 after TMZ, for 24 h. (A) Representative control cells and cells treated with TMZ or TMZ plus senolytics. (B) CellMorph scatterplot. Inserts show CellMorph density plot. cLI, Large Irregular cells (E‐state); cLR, Large Regular cells. (C) Percentage of cells at cLR and cLI. (D) Mean cell area and CII. (E) Number of cells per field relative to TMZ (considered 100%).

These findings are clinically relevant because senescence levels are related to poor prognosis in cancer, including glioblastoma (Li et al. 2022; Salam et al. 2023; Bao et al. 2024; Santos‐Sousa et al. 2025). Therefore, inducing senescence to block tumor growth followed by eliminating SnCs is a promising strategy. Thus, we evaluated the sensitivity of TMZ‐induced SnCs to senolytics (Dasatinib—target multiple kinases, Navitoclax—target Bcl‐xL, ABT‐737—target Bcl‐xL and Bcl‐2, and A‐1210477—target MCL‐1) (Zhu et al. 2016; Kudlova et al. 2022), focusing on the differential response of SnCs harboring distinct morphofunctional states. Dasatinib reduced cell size compared to TMZ alone, increasing shrunk round‐shaped cells (Figure 6A). CellMorph confirmed the reduction of cells in cLI (E‐state cells) and cLR (F‐state cells) quadrants (Figure 6B,C). Furthermore, adding Dasatinib reduced cell area (Figure 6D), CII (Figure 6D) and cell number compared to TMZ (Figure 6E). In contrast, the other senolytics had no or little effect on TMZ‐treated cells. The main results of cell morphometry were corroborated by NMA, which confirmed that Dasatinib caused nuclear shrinkage (Figure S11A–C), reduction in nLR (Figure S11B,D), the mean nuclear area (Figure S11C) and LR nuclei (Figure S11D). NMA also confirmed the reduction of nuclei per field (Figure S11E) compared to TMZ alone. As demonstrated in Figure S12, there is no effect of any tested senolytic drug on shape descriptors and distribution of LR or LI cells in untreated cells. However, representative images (Figure S12A) show slight variations in overall cell shape and substantial heterogeneity among cells, resulting in a wide range of standard deviations in the metrics. One noticeable effect of senolytic over general cell population is demonstrated in Figure S12E, where cells appear to have boosted proliferation, which can be due to the elimination of any senescent cell group among the overall population.

## 3 Discussion

In this study, we comprehensively characterize the heterogeneity and plasticity of glioblastoma cells induced to senescence by TMZ. Through an experimental design resembling the clinical schedule, our study introduced novel insights into the states and plasticity of SnCs, including (a) the emergence of two main morphofunctional states for enlarged cells, (b) long‐lasting autophagy in cells progressing along the senescent phenotype, (c) differences in cells living in each morphofunctional state, including levels of autophagy, p16 and Bcl‐2 antiapoptotic proteins, (d) the differential sensitivity to autophagy modulators and senolytics by TMZ‐induced SnCs, and (e) the phenotypic dynamics of SnCs.

The enrichment of SnCs is a hallmark of human cancers (Hanahan 2022). Clinically, increased scores of senescence are associated with worse prognosis (Santos‐Sousa et al. 2025), including in glioblastoma (Gnanavel et al. 2021; Liu et al. 2023; Bao et al. 2024). Therefore, understanding morphofunctional features of senescent cancer cells, mainly poorly understood phenotypes such as phenotypic heterogeneity and plasticity, is clinically relevant (Schmitt et al. 2022). Recent findings have shown different layers of heterogeneity, including variations of SnCs inside the same population, from different tissues, or induced by different stresses (Basisty et al. 2020; Beck et al. 2020; Cayo et al. 2021; Wiley and Campisi 2021; Troiani et al. 2022; Bitencourt et al. 2024). However, most evidence regarding intercellular heterogeneity in SnCs comes from transcriptomic studies, while data about the morphofunctional dynamics and heterogeneity are lacking (Kirschner et al. 2020; Duran et al. 2024). Our findings confirm that cells induced to senescence by TMZ are heterogeneous in morphological, metabolic and molecular features. Furthermore, the progression throughout the senescent phenotype was also heterogeneous. Likewise, a few studies have also observed variation in classic markers of senescence, such as cell shape and size (Kamat et al. 2024), p16 expression levels (Wong et al. 2019; Zhao et al. 2024) and SA‐β‐gal activity (Ashraf et al. 2023).

We provided a detailed characterization of TMZ‐induced enlarged cells, suggesting the enrichment of two cell morphofunctional states, E‐state and F‐state. These states had widely different morphometries and activation of cell survival mechanisms, leading to differential sensitivity to autophagy inhibitors and senolytics. Early autophagy inhibition enriched E‐state cells and decreased the number of F‐state cells with no significant toxicity, suggesting a role for autophagy in senescence progression. This data corroborates previous findings showing increased autophagy in SnCs (Carroll et al. 2017; Wiley and Campisi 2021; Mansfield et al. 2024), contributing to apoptosis resistance (Kucheryavenko et al. 2019; L'Hôte et al. 2021; Beltzig, Schwarzenbach, et al. 2022; Bientinesi et al. 2024). Furthermore, the differential effect of early autophagy inhibition in E‐ or F‐state cells is relevant since autophagy seems to be important for SASP production (Laberge et al. 2015; Cayo et al. 2021), and enlarged cells with open chromatin, which resemble F‐state cells, produce greater amounts of SASP (Hao et al. 2022). Therefore, early autophagy inhibition could act both as a senopreventive and a senomorphic strategy, avoiding the phenotypic progression of SnCs and mitigating the pro‐tumor effects played by those cells in the tumor microenvironment (TME), respectively. Complementary to this, HCQ, a late‐phase autophagy inhibitor, had a senolytic effect, as previously reported (L'Hôte et al. 2021), but without specificity for one of the SnC morphostates. The combination of autophagy inhibition with chemotherapeutic agents has been widely reported to yield beneficial effects, with the timing of autophagy inhibition being critical to affect the fate of cancer cells (Filippi‐Chiela et al. 2015; Vargas et al. 2020; Baldasso‐Zanon et al. 2024). Notably, previous studies investigating the effects of early autophagy inhibition (e.g., using 3‐MA) in glioblastoma models have primarily focused on short‐term responses, leaving a significant gap regarding the long‐term impact of such intervention on glioblastoma regrowth (Lin et al. 2012; Filippi‐Chiela et al. 2015; Chio et al. 2018). Likewise, the long‐term consequences of early autophagy inhibition in the phenotypic progression and dynamics of E‐ or F‐state cells remain unclear. Given that tumor recurrence is a frequent scenario in glioblastoma, long‐term experiments including early autophagy inhibitors may reveal both the impact of this modulation on the phenotypic plasticity of senescent cells and on tumor regrowth.

While the impact of SnCs in modulating the phenotypic plasticity of other cells is well described (Silva et al. 2024), the phenotypic plasticity of SnCs is even less known than morphofunctional heterogeneity (Hanahan 2022). To shed light on that, we tracked individual SnCs and observed that the same SnC occupies different phenotypic spaces over time. We found that the progression from E‐state to F‐state was more frequent, but transitions from F‐state to E‐state also occurred. Tracking individual SnCs over time serves as proof‐of‐principle of their phenotypic plasticity, adding a new layer of complexity to the biology of senescence. This observation also implies that SnCs can adapt to environmental or intracellular stresses by changing anti‐apoptotic proteins' expression or autophagy (Ben‐Porath and Weinberg 2004; Sah et al. 2021; Cassidy and Narita 2022), impacting their sensitivity to senotherapies (Soto‐Gamez et al. 2019; Deryabin et al. 2021). Although we observed cells with a phenotype similar to the control at all time points after the TMZ treatment, the origin of the cells that promoted population regrowth remains uncertain and requires further study. Among the hypotheses that would explain its presence in the population are (a) resistant cells capable of avoiding the damage caused by TMZ and, consequently, senescence; (b) cells originating from an abnormal division process of senescent cells, such as budding‐like events; (c) cells with senescent characteristics that have returned to proliferate; (d) cells with morphological characteristics of senescence but which were, in fact, quiescent or dormant in the initial days after treatment, and which regained proliferative capacity. Although our data consistently demonstrate morphological heterogeneity in cells with senescent characteristics across the analyzed glioblastoma cell lines and primary culture, the biological origin of cell morphotypes remains uncertain. Considering the transcriptome, virtually all studies agree that senescent cells are heterogeneous with respect to levels of senescence markers (Tang et al. 2019; Neri et al. 2025), SASP constitution (Hernandez‐Segura et al. 2017; Bitencourt et al. 2024), and survival pathways (Troiani et al. 2022). Nevertheless, this evidence illustrates the transcriptomic heterogeneity of senescent cells, but it does not advance in proving the biological origins of this variability. In this scenario, Zheng and colleagues recently demonstrated that the transcriptomic profile associated with the senescent cell cycle phase is an important factor in defining the SASP composition and response to the senolytic ABT263 (Neri et al. 2025). Another variable that could promote the morphofunctional heterogeneity we observed is cellular metabolism. Although sensitivity to senescence induced by oxidative stress is indeed influenced by the metabolic status of cells at the time of stress (Chen et al. 2025), it remains unclear whether this status mediates the heterogeneity of senescent cells, especially with respect to morphological states. On the other hand, although subcellular characteristics associated with cell morphology, such as cytoskeletal morphofunctionality and plasma membrane constitution, are altered in senescent cells when compared to proliferative parental cells (Bitencourt et al. 2024), their variability among senescent cells has not been characterized. Nevertheless, given that we observed morphofunctional transitions to E‐state and F‐state cells, we can infer that, as important as defining the biological origins of the observed heterogeneity is, understanding the molecular players involved in both the transitions between senescent cell states and the progression of the phenotype is equally important. While for some senescent cell states, such as those related to the immune system or the secretory state, the intracellular pathways and transcription factors involved are already known (De Mendonça et al. 2025), the characterization of the heterogeneity and morphofunctional dynamics of senescent cells remains incipient. It is possible that factors such as the phase of the cell cycle at which senescent signaling is activated, cellular metabolism, and the ability to repair and respond to cellular damage are associated with the predominant morphofunctional state of the senescent cell. This is because the well‐established relationship between form and function in biology suggests that the acquisition and maintenance of the E‐state or F‐state are associated with distinct impacts on the microenvironment in which senescent cells are found.

The different senescent states were also differentially sensitive to classic senolytics. Dasatinib, which blocks the activity of several tyrosine kinases, mainly sensitized E‐state cells. In contrast, both E‐state and F‐state cells were resistant to senolysis induced by molecules targeting Bcl‐2 proteins, possibly due to the compensatory and redundant roles of anti‐apoptotic proteins of the Bcl‐2 family (Eichhorn et al. 2014; Carrington et al. 2017). Thus, inhibiting only one or two Bcl‐2 proteins was insufficient to trigger senolysis, which reinforces the need for better characterization of SnC heterogeneity for clinical purposes. However, although the expression of survival proteins by SnCs is heterogeneous, as shown by transcriptomic data (Saul and Kosinsky 2021), it is uncertain whether the same SnC expresses high levels of more than one member of the Bcl‐2 family. Although we found a senolytic effect for Dasatinib, a significant proportion of senescent cells survived. In this context, and based on evidence suggesting that the same senescent cell can overexpress different cell survival proteins (Troiani et al. 2022), rational tests combining different senolytics with complementary targets should be prioritized in order to enhance the elimination of senescent cells.

Our data reveals that senescent subpopulations defined by distinct morphofunctional phenotypes present different status regarding the FUCCI cell cycle marker. While F‐state cells are predominantly G1‐FUCCI cells, E‐state cells are distributed between cells with G1 and G2 molecular features, with a modest enrichment in G2‐FUCCI relative to the whole population of enlarged cells. This observation aligns with accumulating evidence that, molecularly, cellular senescence is not restricted to cells with a gene expression pattern typical of G1 but can also be established and maintained in cells with G2 molecular features, particularly in DNA damage–induced senescence (Mao et al. 2012; Ye et al. 2013; Restall et al. 2015). Furthermore, E‐state cells had higher levels of p16 and CDKN2A promoter activity than F‐state cells. These results agree with data from fibroblasts and endothelial cells induced to senescence by irradiation, in which senescent cells harboring a G2 transcriptomic profile exhibited higher levels of senescence markers (including increased p21, HMGB1, and IL‐6, and reduced LaminB1) compared to senescent cells with a G1 transcriptomic profile (Neri et al. 2025). Within this framework, the preferential association of the E‐state with G2 markers supports the hypothesis that early or intermediate senescent phenotypes may arise prior to mitotic entry, whereas the accumulation of F‐state cells with a G1 profile may reflect a more terminal phase of cell‐cycle withdrawal. Importantly, our observation that F‐state cells with G1 features are larger than those with G2 features further suggests that morphometric remodeling accompanies cell‐cycle transitions, consistent with a progressive reinforcement of the senescent phenotype.

Mechanistically, several studies have demonstrated that senescent cells initially with G2 status can subsequently undergo mitotic bypass or “mitotic slippage,” resulting in a G1 phenotype characterized by sustained CDK inhibition and RB pathway engagement (Hedblom et al. 2013; Gire and Dulić 2015). In this context, a recent study showed that in the late stages of TMZ‐induced senescence, glioblastoma cells can reinitiate DNA synthesis and undergo endoreplication, thereby contributing to polyploidization and increased cell size. This process was determined by the p21CIP1–CDK1/2 axis, which is sufficient to enforce durable cell‐cycle arrest (Schwarzenbach et al. 2025). These findings provide a mechanistic basis for the establishment of senescence in cells in G2 following TMZ‐induced DNA damage and support the possibility that progressive CDK1/2 inhibition enables the subsequent acquisition of G1 molecular features without productive mitotic entry. This switch from G2‐to‐G1 molecular profile offers a plausible explanation for the phenotypic progression observed in our model, in which E‐state cells precede and give rise to F‐state cells over time. Our single‐cell tracking data directly support this dynamic relationship, reinforcing the concept that senescent states are not static endpoints but evolve through coordinated changes in the expression of cell‐cycle control genes, cell‐cycle‐dependent enzymatic activity, and cellular architecture. Recent studies further indicate that senescent cells arising from cells with a transcriptomic signature of G2 have distinct functional outputs, including differences in SASP composition, stress‐response signaling, and sensitivity to senolytic agents (Feringa et al. 2018; Neri et al. 2025). Complementarily, other models propose progression from proliferation to quiescence (reversible arrest), from which cells may or may not transition into irreversible senescence (Ashraf et al. 2023; Fernandez et al. 2025). Integrating these concepts with our morphofunctional analysis, it is plausible that cells arrested in G2 following TMZ‐induced DNA damage initially adopt an E‐state, followed by progressive cell enlargement towards the F‐state (i.e., transitioning from cN through cLI to cLR). During this process, mitotic slippage may occur, enabling the acquisition of a G1‐phase molecular profile. In parallel, G1‐damaged cells may arrest at G1 and undergo size increase, following a trajectory from cN to cLR. Taken together, our findings integrate morphofunctional heterogeneity with cell‐cycle molecular status and support a model in which senescence unfolds through a structured phenotypic progression, linking G2‐associated E‐state cells to G1‐enriched F‐state cells as part of a dynamic molecular continuum. Furthermore, although our evidence from tracking individual cells and specific proliferation markers suggests that large cells are not proliferative, it is not possible to completely guarantee that these cells will never trigger any process associated with cell division like endoreplication (Zeng et al. 2023), endomitosis (Zhang et al. 2023), mitotic slippage (Ye et al. 2013), or even the generation of daughter cells from complex and poorly understood mechanisms such as budding‐like events (Czarnecka‐Herok et al. 2022).

The U87 MG cell line (U87 MG, ATCC HTB‐14) has long been recognized as a source of molecular inconsistencies (Allen et al. 2016; Bairoch 2018). This is partly attributable to its uncertain origin—although initially described as a glioma derived from Uppsala University, subsequent molecular analyses demonstrated that the original Uppsala tumor and the currently distributed ATCC U87 MG line represent distinct cell lines (Allen et al. 2016). ATCC therefore classifies U87 MG HTB‐14 as being of unknown origin, most likely derived from a male glioblastoma. This historical misidentification contributes to the conflicting biological data reported. Classically, U87 MG has been described as harboring a homozygous deletion of the CDKN2A locus, resulting in loss of p^16INK4a^ expression (Srivenugopal and AliOsman 1997; Clark et al. 2010; Michaud et al. 2010; Wolter et al. 2022). However, a growing number of reports challenge this assumption. Recent experimental evidence has demonstrated basal p16 protein expression in U87 MG cells, which necessarily implies the presence of at least one functional CDKN2A allele (Boukhari et al. 2015). In addition, combined RT‐qPCR, immunoblotting, and CpG methylation analyses further support the existence of residual CDKN2A gene copies and detectable basal p16 expression in U87 MG cultures (Wang et al. 2024). Our data are consistent with this evidence, demonstrating baseline expression in U87 cells, with a significant increase in expression after treatment with TMZ. Collectively, these findings suggest that the p16 status of U87 MG cannot be considered universally deficient and that sublines retaining CDKN2A expression likely exist, in accordance with our data.

Notably, the evidence of morphofunctional heterogeneity observed in senescent cells, as well as their responses to autophagy and senolytic modulators, applies to molecular scenarios involving cells carrying functional TP53 and p16. TP53 mutations occur in approximately 25%–30% of primary glioblastomas, while CDKN2A/B homozygous deletion occurs in roughly 40%–60% of cases (Brennan et al. 2013). In this scenario, our group previously reported that senescence is a major outcome in TP53‐ and p16‐proficient glioblastoma cells, whereas TP53‐mutated cells often exhibit alternative outcomes, such as apoptosis or mitotic catastrophe (Filippi‐Chiela et al. 2013, 2015; Silva et al. 2016). These studies added to evidence from the literature confirming the dependence of TP53 on the duration of G2/M cell arrest and senescence induction by TMZ, not only in glioblastoma cells (Hirose et al. 2001; Wark et al. 2024) but also in melanoma (Mhaidat et al. 2007). In this context, inhibition of the cell cycle arrest pathway, including inhibition of Chk1 or E6‐mediated TP53 deficiency, increases the toxicity of TMZ by attenuating cellular senescence and diverting cells towards mitotic catastrophe (Hirose et al. 2005; Wark et al. 2024).

The heterogeneity and plasticity of glioblastoma cells induced to senescence by TMZ add new layers of difficulty to the success of senotherapies not only for glioblastoma but also for other cancers, since heterogeneity and plasticity are critical factors for prognosis (Dagogo‐Jack and Shaw 2018; Hanahan 2022; Cordani et al. 2024). Therefore, our evidence opened new alternatives for two‐step strategies aiming to attenuate the pro‐tumor effects of cellular senescence (Sieben et al. 2018) through the rational combination of chemotherapeutics with autophagy modulators or the expanding field of senotherapies.

## 4 Material and Methods

### 4.1 Cell Culture

U‐87 MG and A172 cells were purchased from ATCC (Rockville, MD) and maintained in DMEM low glucose supplemented with 10% fetal bovine serum (FBS), 1% penicillin/streptomycin, and 0.1% Amphotericin B, at 37°C and 5% CO_2_ in a humidified incubator. Primary GBM culture was established from a biopsy of a GBM tumor following the ethical procedures approved by the Ethical Committee of PUC‐RS number 07/03562, and cultured as previously described.

### 4.2 Cell Engineering

U‐87 cells stably expressing GFP‐LC3 and mApple‐53BP1trunc, U‐87 cells stably expressing mKO2‐hCDT1 and mAG‐hGEM (FUCCI) and A172 cells stably expressing CDKN2A promoter (908 bp) controlling GFP expression and nuclear signaling (Fluosen) were generated by lentiviral transduction. Lentiviral particles were produced by co‐transfecting HEK‐293 cells with reporter plasmids Apple‐53BP1‐trunc (Addgene #69531) and GFP‐LC3 (Vectorbuilder VB200313‐2861jfb), or mKO2‐hCDT1 and mAG‐hGEM (Addgene #86849) or CDK2NA promoter/GFP/nuc (Vectorbuilder VB231218‐1589gjp) and third‐generation lentiviral vectors (pMDLg/pRRE [Addgene, 12251], pMD2.G [Addgene, 12253]), and pRSV‐Rev (Addgene, 12259) using linear polyethylenimine (PEI) 25,000 (Polysciences, 23966). Viral‐containing supernatant was collected 4 and 5 days post‐transfection, filtered through a 45 μm syringe filter, and stored at −80°C. For transduction, cells were plated in 24‐well plates and incubated with 250 μL of each viral suspension and 8 μg/mL polybrene per well. Cells were centrifuged at 700 × *g* for 45 min at 25°C, and after 24 h, the medium was replaced. Stable cell lines were selected by incubating the cells in medium containing 5 μg/mL puromycin (Sigma‐Aldrich, P8833) for 3 days. All cells were maintained in DMEM supplemented with 10% Fetal Bovine Serum, 1% penicillin and streptomycin, and 0.1% amphotericin B in 5% CO_2_ at 37°C.

### 4.3 Cell Sorting

U‐87 cells stably expressing GFP‐LC3 and mApple‐53BP1trunc were selected for intermediary fluorescence levels of both reporters through cell sorting at BD FACS Melody. Cells were grown upon reaching 80% confluence and prepared into a 1 × 10^5^ cells/mL DMEM solution. Populations positive for BL1 (GFP‐LC3) and BL3 (mApple‐53BP1trunc) filters were sorted at 100 cells/well into a 96‐well plate. Cells were grown until reaching an 80% confluence and then subcultured to a 6‐well plate. Fluorescence testing was performed at the Incucyte S3 system, and the well representing a population with intermediary levels of both fluorescences was used.

### 4.4 Drug Treatments

Temozolomide (TMZ, Sigma‐Aldrich, T2577) treatment was conducted at 50 μM for 5 days, followed by medium removal and growth in drug‐free medium (DFM) for 21 days or as specified in each experiment. Early autophagy inhibitor 3‐methyladenine (3MA, Sigma‐Aldrich M9281) was used at 5 mM for 1 h at Day 5 and Day 6 after TMZ. Late autophagy inhibitor hydroxychloroquine (HCQ, Sigma‐Aldrich, H0915) was used at 40 μM for 24 h. Senolytics treatment was conducted as follows: Dasatinib at 1.5 μM (MedChemExpress, HY‐10181; target: multiple kinases), Navitoclax at 1 μM (MedChemExpress, HY‐10087; targets: Bcl‐xL), ABT‐737 at 1 μM (MedChemExpress, HY‐50907; targets: Bcl‐xL and Bcl‐2), and A‐1210477 at 13 nM (MedChemExpress, HY‐12468; targets: Mcl‐1). All senolytics were maintained for 24 h.

### 4.5 Cumulative Population Doubling (CPD)

Cell counting was performed in a Neubauer. At indicated days, the number of cells and the CPD were determined, as previously described (Silva et al. 2016), according to the equation PD = [ln *N*(*t*) − ln *N*(*t*o)]/log_2_, where *N*(*t*) is the number of cells per well at the time of the cell counting (passage) and *N*(*t*o) is the initial number of seeded cells. The sum of PDs was then plotted over time to assess the long‐term proliferation profile through CPD.

### 4.6 Autophagy Assessment

Autophagy was measured through the GFP‐LC3 marker. Briefly, the percentage of cells with at least 5 clear green dots in the cytoplasm was determined for at least 100 GFP‐LC3 cells per picture, in 5 pictures (Klionsky et al. 2021; Liu et al. 2023). We also measured the GFP‐LC3 area using Image Pro Plus 6.0 software (Media Cybernetics, Silver Spring, MD, USA). These data were plotted with the nuclear and cell areas on a contour plot graph using SigmaPlot software (Systat Software Inc., San Jose, CA, USA). The area of green dots in the cytosol was measured by adjusting the threshold in ImageProPlus. The total autophagosome area was acquired by the sum of individual autophagosomes' areas of each cell.

### 4.7 Nuclear Morphometric Analysis (NMA)

The nuclear morphometry was analyzed using the NMA developed by our group (Filippi‐Chiela et al. 2012). Briefly, images were taken in the Incucyte S3 system, followed by nuclear segmentation and data acquisition using Image Pro Plus 6.0 software (Media Cybernetics, Silver Spring, MD, USA) or ImageJ plugin (www.ufrgs.br/labsinal/nma). Data are presented as a plot of Nuclear Area versus nuclear irregularity index (NII) (Figure S1A). In this study, the relevant quadrants of NMA are: Normal (nN), Large Regular (nLR), and Small Regular (nSR). “n” here is used to refer to nuclei, not to be confused with cellular (c) morphometry, as described in the next section. Briefly, the NMA graph has a region of normal nuclei (N), and 5 other regions that classify individual nuclei according to shape and size (Figure S1A). Representative nuclei are shown in Figure S1A. We also show NMA data as a contour plot graph using SigmaPlot (Systat Software Inc., San Jose, CA, USA).

### 4.8 Cellular Morphometric Analysis (CellMorph)

Cellular morphometry was analyzed using CellMorph, developed by our group (Quaiato De Oliveira et al. 2025). To that, individual cells were segmented using Image Pro Plus 6.0. The protocol and graphic solution for CellMorph is like NMA (Figure S1B). It classifies individual cells based on cell area and shape, measured by Cellular Irregularity Index (CII). In this study, the relevant quadrants for cellular morphometry are: Normal (cN), Large Regular (cLR), and Large Irregular (cLI). The “c” before each category represents “cells,” to differentiate NMA (n) from CellMorph (c). Representative cells are shown in Figure S1B.

### 4.9 Senescence Assessment

Senescence was quantified by incubating U‐87 WT cells with 5‐dodecanoylaminofluorescein di‐beta D‐galactopiranoside (C12‐FDG, Life Technologies, D2893) (Cahu and Sola 2013), a fluorescent substrate of the Senescence‐Associated β‐Galactosidase (SA‐β‐gal). To that, cells were incubated with a solution containing C12‐FDG 33 μM and DMEM media for 2 h at 37°C. Cells were then trypsinized and analyzed in Attune Nxt (ThermoFisher).

For x‐gal staining, cells were fixed using paraformaldehyde 4% (Sigma‐Aldrich, 1.00496) 20 min at 37°C, washed 1× with PBS, and permeabilized with Triton X‐100 0.1% (Sigma‐Aldrich, 93443) for 5 min at room temperature. Cells were incubated with fresh SA‐β‐gal staining solution [1 mg/mL X‐gal substrate (Sigma‐Aldrich, 3117073001), 40 mM citric acid/sodium phosphate (pH 6.0), 5 mM potassium ferrocyanide, 5 mM potassium ferricyanide, 150 mM NaCl, and 2 mM MgCl] for 6 h at 37°C. Then, cells were stained with a solution containing 300 nM DAPI and 0.1% Triton X‐100 (v/v in PBS) for 30 min at room temperature.

### 4.10 Proliferation Assay (CFSE Dilution)

Carboxyfluorescein succinimidyl ester (CFSE) was used to determine proliferation (Terrén et al. 2020). Briefly, cells were incubated with CFSE at 10 μM in PBS for 20 min at 37°C and 5% CO_2_ humidified incubator, 3 days prior to analysis. After, CFSE solution was retrieved and fresh media was added to the cells. On the indicated day, cells were fixed using paraformaldehyde at 4% for 20 min at 37°C, then washed once with PBS 1× and imaged. Fluorescence decay is correlated to proliferation and division.

### 4.11 Cell Imaging (Including Live Cell Imaging) and Cellular Segmentation

Images of fluorescent and bright‐field cells were acquired in an Incucyte S3 (Sartorius). Images of bright‐field cells stained for SA‐β‐gal activity with x‐gal, CFSE, or immunostained were acquired with an Olympus IX71 microscope.

### 4.12 Immunocytofluorescence

Cells were plated over a poly‐L‐lysine (Sigma‐Aldrich, P8920) 0.01% coated 22 × 22 mm cover glass (Olen, BR723015) on a 24‐well plate (BCL2 family and p16) or directly on the wells (pH 3). Cells were fixed using paraformaldehyde 4% for 20 min at 37°C. Cells were washed once with PBS 1× and permeabilized with Triton X‐100 0.1%. Protein blocking with 3% BSA was performed for 1 h at room temperature. Primary antibody incubation was overnight at 4°C, using the determined antibody (Cell Signaling, 2764) at 1:100, or 1:800 for anti‐pH 3. Incubation with a secondary anti‐rabbit IgG labeled with FITC (Cell Signaling, 2764), at 1:2000, or 1:1600 for anti‐rabbit IgG against anti‐pH 3, for 1 h 30 min at room temperature. The slides were mounted with Fluoroshield (Sigma‐Aldrich, F6182) mounting medium with DAPI or wells were imaged directly (for p‐H3). Fluorescence intensity was measured using ImageJ software through the threshold method as described (Shihan et al. 2021) when indicated. Total cell fluorescence was obtained by multiplying cell area and MFI variables. We also acquired the information about the Circularity of individual cells, given by the following equation: (4π*(area/perimeter^2^)). Circularity values range from 1 (a perfect circle) to zero (for elongated polygons). Cells with circularity below or above the median were classified as low‐circularity (E‐state phenotype) and high‐circularity (F‐state), respectively. For p‐H3 analysis, parameters were extracted from the bright‐field images for CellMorph evaluation, and cells were counted as positive or negative depending on the fluorescence presence or absence.

### 4.13 Flow Cytometry

All flow cytometry experiments were performed using Attune NxT flow cytometer (Thermo Fisher). For P16 expression assessment (Fluosen), A172 engineered cells were TMZ treated as previously described and cells were then trypsinized and analyzed at indicated days. As a control evaluation, for endogenous p16 expression, A172 wt cells were fixed using paraformaldehyde 4% for 15 min, washed with PBS 1× and permeabilized with Triton X‐100 1% for 30 min. Protein blocking was conducted with 1.5% FBS for 20 min. Then cells were incubated with anti‐p16 1:50 (Abcam ab54210) for 30 min at 4°C then washed with PBS and incubated with goat anti‐mouse IgG (Invitrogen A‐11005), at 1:200, for 30 min at room temperature.

Cell cycle analysis was performed by trypsinizing cells at indicated times and fixing cells using ethanol 70% added under vigorous agitation at vortex then left at 4°C for 2 h. Once fixed, cells were centrifuged at 1500 rpm for 5 min. Cells were resuspended in PBS 1× at a concentration of 200 cells/L. Cells were then centrifuged again and resuspended at a solution containing Triton 0.1%, RNAse 40 g/mL and Propidium Iodine 12 g/mL, and analyzed at cytometer. Wt cells were used as a non‐fluorescent normalization.

### 4.14 Statistics

All experiments were performed at least three times independently. The normality of the groups was assessed using the Shapiro–Wilk test. For comparing means in samples with normal distribution, we conducted *t*‐test (for 2 groups) or analysis of variance (ANOVA) (for 3 or more groups), followed by a Tukey test. For samples with abnormal distribution, we use the Mann–Whitney test. For multiple comparisons of groups with abnormal distribution, we use the Kruskall–Wallis test with Bonferroni correction. The chi‐square test was used to compare proportions. Analyses were performed using SPSS 18.0. Unless otherwise indicated, normally distributed data are shown as mean ± standard deviation, while abnormally distributed data are shown as median ± interquartile range (IQR). All *p*‐values under 0.05 were considered significant. In all figures, * indicates *p* ≤ 0.05, ** indicates *p* ≤ 0.01, and *** indicates *p* ≤ 0.001.

## Author Contributions

S.A.R., H.Q.O. contribute equally to this study. S.A.R., H.Q.O. and E.C.F.‐C. contributed to the study conception and design. Material preparation, data collection and analysis were performed by S.A.R., H.Q.O., E.C.F.‐C., L.B.M., N.B.O., M.C.J., D.S.‐S., F.S.‐C., L.L., L.C.P., F.D.P.O., M.L., G.L. and E.C.F.‐C. The first draft of the manuscript was written by S.A.R., H.Q.O. and E.C.F.‐C. and all authors commented on previous versions of the manuscript. All authors read and approved the final manuscript.

## Funding

This work was supported by Conselho Nacional de Desenvolvimento Científico e Tecnológico (CNPq/MCTI/FNDCT No. 18/2021—Universal), Fundação de Amparo à Pesquisa do Estado do Rio Grande do Sul (EDITAL FAPERGS 07/2021—PROGRAMA PESQUISADOR GAÚCHO‐PqG), and Hospital de Clínicas de Porto Alegre (20200497).

## Ethics Statement

All human samples were obtained with informed consent from participants. The study was approved by the Institutional Review Board of PUC‐RS project number 07/03562 and conducted in accordance with the Declaration of Helsinki.

## Conflicts of Interest

The authors declare no conflicts of interest.

## Supporting information


**Figure S1:** Overview of Nuclear Morphometric Analysis (NMA) and Cellular Morphometric Analysis (CellMorph). (A) NMA graph. The classic scatterplot of Nuclear Area versus Nuclear Irregularity Index (NII). We upgraded quadrants and abbreviatures, subdividing the nLR area in two, based on the number of standard deviations (SD). (A—right) Nuclei representing the phenotypes of interest in this study. (B) CellMorph graph. The classical scatterplot of Cell Area versus Cellular Irregularity Index (CII). We upgraded quadrants and abbreviatures, subdividing the cLR area in two, based on the number of standard deviations (SD). cLI represents Large and Irregular cells. (B—right) Cells representing the phenotypes of interest in this study.

**Figure S2:** Morphofunctional states of enlarged cells in A172 and primary glioblastoma (pGB) cells treated with TMZ. A172 and pGB cells were treated with TMZ 50 μM for 5 days, followed by reseeding in Drug‐Free Medium. Cells were imaged and analyzed after 5 days. (A) Representative images (brightfield) of control and TMZ‐treated A172 cells. (B) CellMorph graphs of A172 cells (control—left; TMZ—right). Density plots are shown as inserts. The red letters represent the cells indicated in figures in (A). (C) Percentage of cells in each quadrant of CellMorph. (D) Representative images of pGB (1 field for control condition; 3 fields for TMZ‐treated cells). (E) x‐gal staining of pGB cells. Representative examples of E‐state (a) and F‐state (b) cells are shown in detail. These cells are also indicated in the CellMorph graph in (F). (F) CellMorph graphs of pGB cells (control—left; TMZ—right). Density plots are shown as inserts. The red letters represent the cells indicated in (E). (G) Percentage of cells in each quadrant of CellMorph.

**Figure S3:** TrackingCellMorph for control and TMZ conditions. Individual control and TMZ‐treated cells were tracked and segmented over time. (A) Control cells. (B) TMZ‐treated cells.

**Figure S4:** Cell cycle distribution, cellular and nuclear morphometric features of U‐87‐FUCCI cells. (A) Representative images of control cells. (B) Flow cytometry of U‐87‐FUCCI cells of control condition. Left—cell cycle distribution considering green fluorescence, where the peak on the left (i.e., negative cells) represents the G1 phase. Right—cell cycle distribution considering red fluorescence, where the peak on the left (i.e., negative cells) represents the G2/M phase. (C and D) Nuclear and cellular morphometric features of control and TMZ‐treated cells, respectively, according to FUCCI patterns. *p < 0.05, **p < 0.01, ***p < 0.001.

**Figure S5:** Cell cycle status in E‐state and F‐state cells. U‐87 cells stably expressing the FUCCI reporter were treated with TMZ 50 μM for 5 days, followed by growth in Drug‐Free Medium. Individual cells were analyzed for cell cycle phase, NMA, and CellMorph in an integrated manner, 5 days after replating in DFM. (A) Representative images of control (top) and TMZ‐treated (bottom) U‐87‐FUCCI cells. (B) Cell cycle distribution for control and TMZ‐treated cells. ***p < 0.001 (chi‐squared test). (C) fucciCellMorph multiphenotypic analysis of control and TMZ‐treated cells. Red, yellow, and green markers represent cells in G1, S, and G2/M phases, respectively. The percentages of cells in cLR (F‐state cells) and cLI (E‐state cells) quadrants are also shown. Inserts represent density plots. (D) Specific analysis of G1 and G2 cells in the fucciCellMorph graph. The percentages of cells in cLR (F‐state cells) and cLI (E‐state cells), as well as density plots for each condition, are also shown. (E) Percentage of G1, S, and G2 cells considering E‐state (cLR) and F‐state (cLI) cells. ***p < 0.001 (chi‐squared test). (F) Representative E‐state and F‐state cells. (G) Cell area for G1 or G2/M cells considering E‐state (cLR) and F‐state (cLI) cells. *p < 0.01, **p < 0.01, ***p < 0.001 (Mann–Whitney).

**Figure S6:** TMZ‐treated enlarged A172 and U87 cells are negative for phospho‐HistoneH3(Ser10). A172 and U87 cells were treated with TMZ 50 μM for 5 days, followed by regrowth in Drug‐Free Medium. After 5 days, immunocytochemistry for phospho‐HistoneH3(Ser10) was performed. (A and B) Representative images of control and TMZ‐treated (A) A172 and (B) U87 cells. (C and D) CellMorph of control (left) and TMZ‐treated cells (right) for (C) A172 and (D) U87 cells. Phospho‐HistoneH3(Ser10)‐positive cells are shown in red.

**Figure S7:** CFSE fluorescence is higher in TMZ‐induced enlarged cells. A172 and U87 cells were treated with TMZ 50 μM for 5 days, followed by regrowth in Drug‐Free Medium. After 5 days, cells were incubated with CFSE. After 2 days, images were acquired, followed by cellular segmentation to extract information about cell size and shape, as well as the green fluorescence (density sum). (A and B) Left—Representative images (brightfield and green fluorescence) of control and TMZ‐treated conditions. Right—green fluorescence (density sum) for control and TMZ‐treated cells; data are shown as median ± IQR (interquartile range). (C) CFSE‐CellMorph graph for control and TMZ‐treated conditions in A172 cells. (D) Green fluorescence (density sum) for Normal (N), F‐state and E‐state cells in TMZ‐treated A172 cells; data are shown as median ± IQR (interquartile range). (E) CFSE‐CellMorph graph for control and TMZ‐treated conditions in U87 cells. (F) Green fluorescence (density sum) for Normal (N), F‐state and E‐state cells in TMZ‐treated U87 cells; data are shown as median ± IQR (interquartile range).

**Figure S8:** Autophagy levels are increased during the transition to increased cell size. Autophagosomes area per cell versus cell area. Dashed black line: threshold separating normal and large cells. Red dashed line box—very large cells (i.e., cells with area higher than the average + 6SD, as shown in Figure S1B).

**Figure S9:** Enlarged cells living in F‐state or E‐state differentially increase levels p16. (A) Levels of p16 according to the circularity. Left—the cells were separated according to the circularity into low and high, and then p16 levels were calculated in each group. Right—representative images. (B) p16 levels versus circularity scatterplot.

**Figure S10:** Relationship between CDKN2A promoter activity and morphometry of A172 cells treated with TMZ. A172 cells were transduced with lentivirus to stably express the CDKN2A promoter controlling the expression of EGFP (named FluoSen reporter). EGFP was tagged with three nuclear localization sequences (NLS). (A) Simplified diagram of the fluorescent reporter. A172‐FluoSen cells were treated with TMZ 50 μM for 5 days, followed by reseeding in Drug‐Free Medium. (B) Representative images of control and TMZ‐treated A172‐FluoSen cells. (C) Flow cytometry analysis to measure green fluorescence levels in control and TMZ‐treated cells. (D) Flow cytometry analysis of endogenous p16 levels, measured in the BL3 (red) channel. (E) Representative images of E‐state (top) and F‐state (bottom) cells with nuclear fluorescence representing the activity of the CDKN2A promoter. (F) Differential CDKN2A promoter activity in E‐state and F‐state cells. (G) Top—correlation between CDKN2A promoter activity and Cellular Irregularity Index (CII). Bottom—correlation between CDKN2A promoter activity and Cell Area. (H and I) Cell cycle analysis of A172‐FluoSen cells using propidium iodide (PI) staining. Both histograms for PI (left) and scatterplot for PI versus green fluorescence (right) are shown. (J) Green fluorescence levels in cells in different phases of the cell cycle.

**Figure S11:** Senolitycs have differential effects in TMZ‐treated glioblastoma cells. Cells were treated with senolytics on Day 10 after TMZ, for 24 h. (A) Representative nuclei of control cells and cells treated with TMZ or TMZ plus senolytics. (B) NMA scatterplot. Inserts show the NMA density plot. (C) Top—mean Nuclear Area; bottom—mean NII. (D) Left—the percentage of nLR; Right—mean area of LR nuclei. (E) Nuclei per field (relative to TMZ, considered 100%).

**Figure S12:** Senolytics effects over non‐treated glioblastoma cells. Cells were treated with senolytics on Day 10, for 24 h. (A) Representative control cells and cells treated with senolytics. (B) CellMorph scatterplot. Inserts show CellMorph density plot. cLI, Large Irregular cells (E‐state); cLR, Large Regular cells. (C) Percentage of cells at cLR and cLI. (D) Mean cell area and CII. (E) Number of cells per field relative to Control (considered 100%).

## Data Availability

The datasets generated during and/or analyzed during the current study are available from the corresponding author on reasonable request.
