# Supplementary figures and images for: Morphofunctional Heterogeneity and Plasticity of Glioblastoma Cells Induced to Senescence by Temozolomide
Source: Aging Cell. 2026 Apr 10;25(4):e70477. doi: 10.1111/acel.70477 (PMC13068634; doi:10.1111/acel.70477)

## Nuclear Morphometric Analysis (NMA)

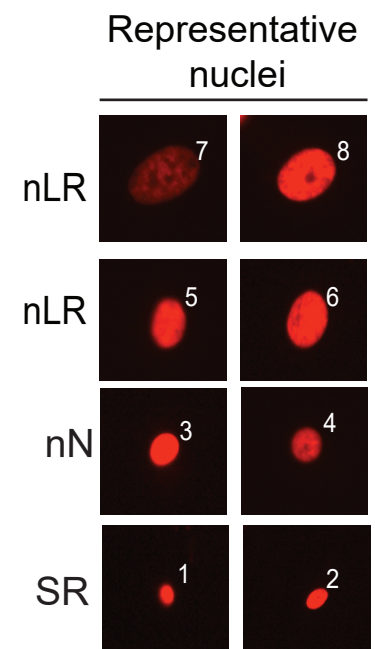

## Cellular Morphometric Analysis (CellMorph)

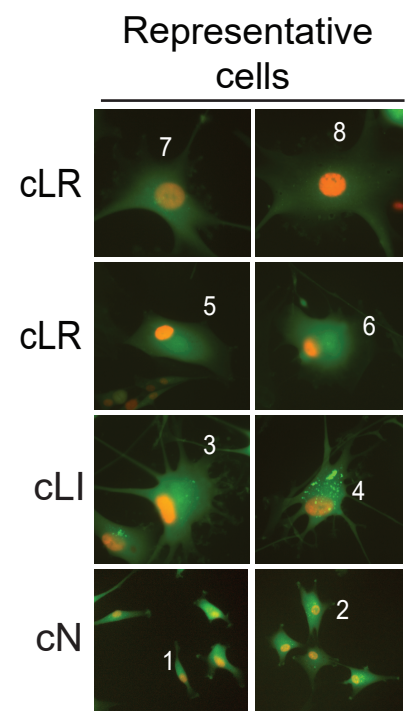

Supplement: Supplementary file 1 — Figure S1: Overview of Nuclear Morphometric Analysis (NMA) and Cellular Morphometric Analysis (CellMorph). (A) NMA graph. The classic scatterplot of Nuclear Area versus Nuclear Irregularity Index (NII). We upgraded quadrants and abbreviatures, subdividing the nLR area in two, based on the number of standard deviations (SD). (A—right) Nuclei representing the phenotypes of interest in this study. (B) CellMorph graph. The classical scatterplot of Cell Area versus Cellular Irregularity Index (CII). We upgraded quadrants and abbreviatures, subdividing the cLR area in two, based on the number of standard deviations (SD). cLI represents Large and Irregular cells. (B—right) Cells representing the phenotypes of interest in this study. [file ACEL-25-e70477-s012.pdf]

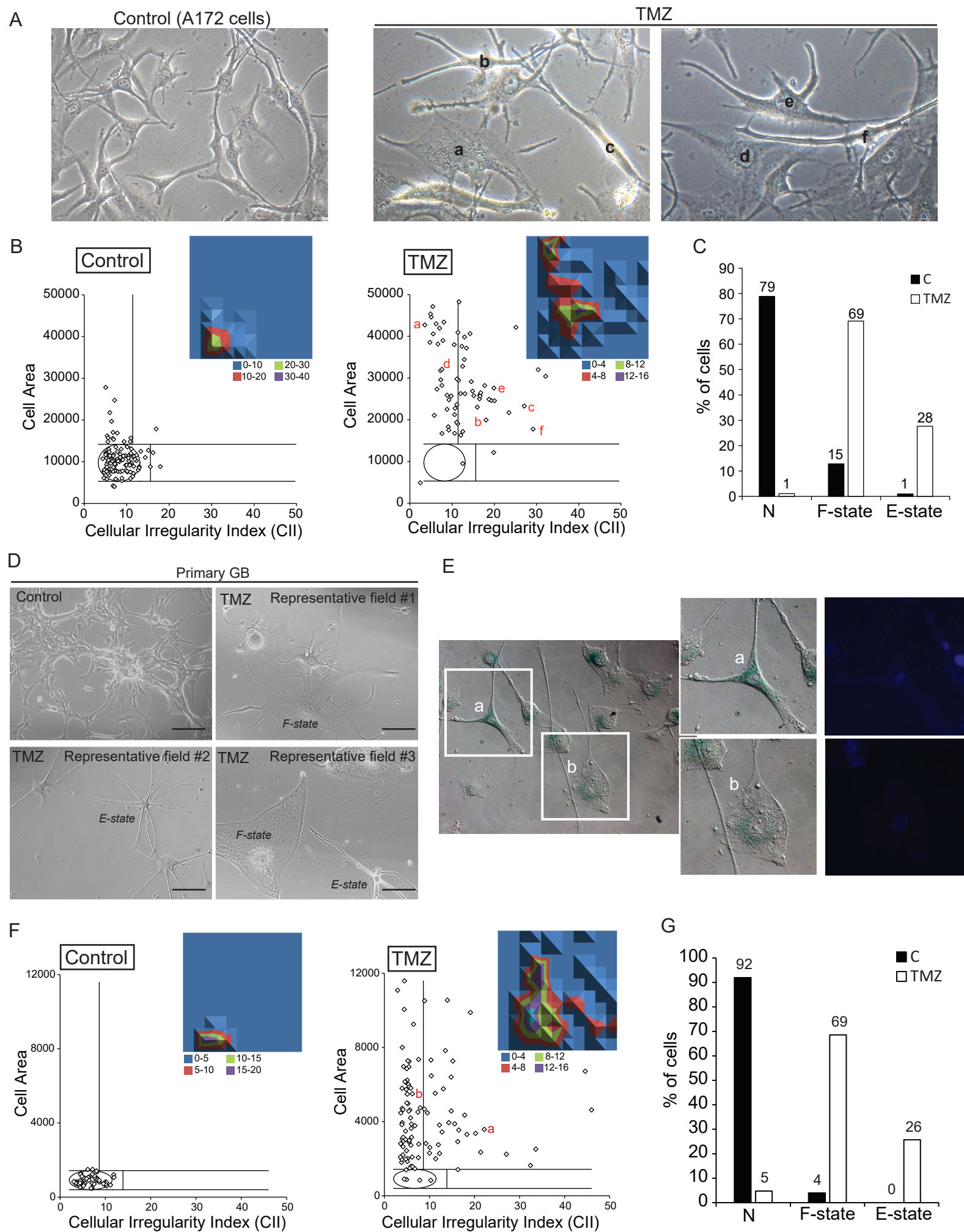

Supplement: Supplementary file 2 — Figure S2: Morphofunctional states of enlarged cells in A172 and primary glioblastoma (pGB) cells treated with TMZ. A172 and pGB cells were treated with TMZ 50 μM for 5 days, followed by reseeding in Drug‐Free Medium. Cells were imaged and analyzed after 5 days. (A) Representative images (brightfield) of control and TMZ‐treated A172 cells. (B) CellMorph graphs of A172 cells (control—left; TMZ—right). Density plots are shown as inserts. The red letters represent the cells indicated in figures in (A). (C) Percentage of cells in each quadrant of CellMorph. (D) Representative images of pGB (1 field for control condition; 3 fields for TMZ‐treated cells). (E) x‐gal staining of pGB cells. Representative examples of E‐state (a) and F‐state (b) cells are shown in detail. These cells are also indicated in the CellMorph graph in (F). (F) CellMorph graphs of pGB cells (control—left; TMZ—right). Density plots are shown as inserts. The red letters represent the cells indicated in (E). (G) Percentage of cells in each quadrant of CellMorph. [file ACEL-25-e70477-s011.pdf]

A

Control

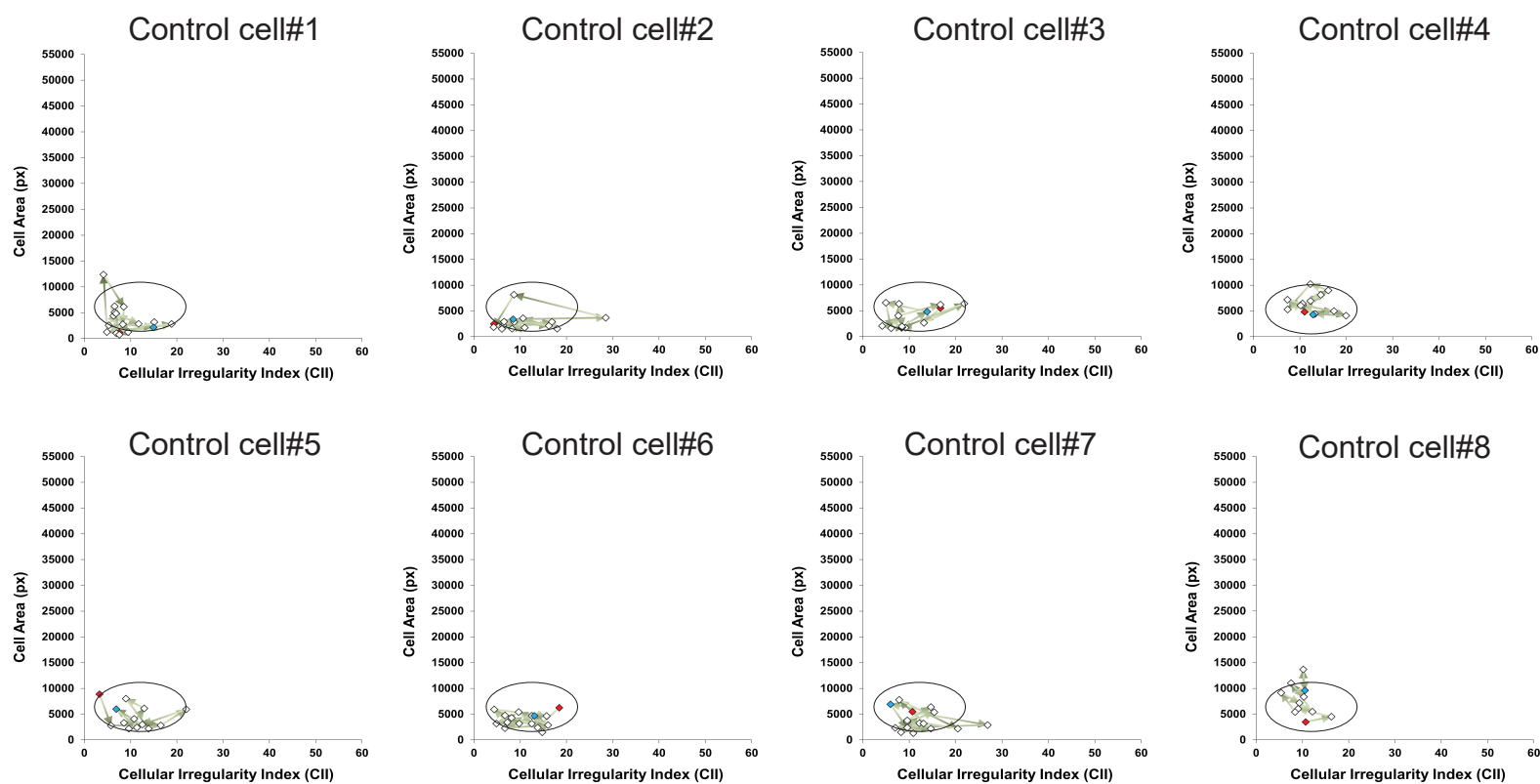

B

TMZ

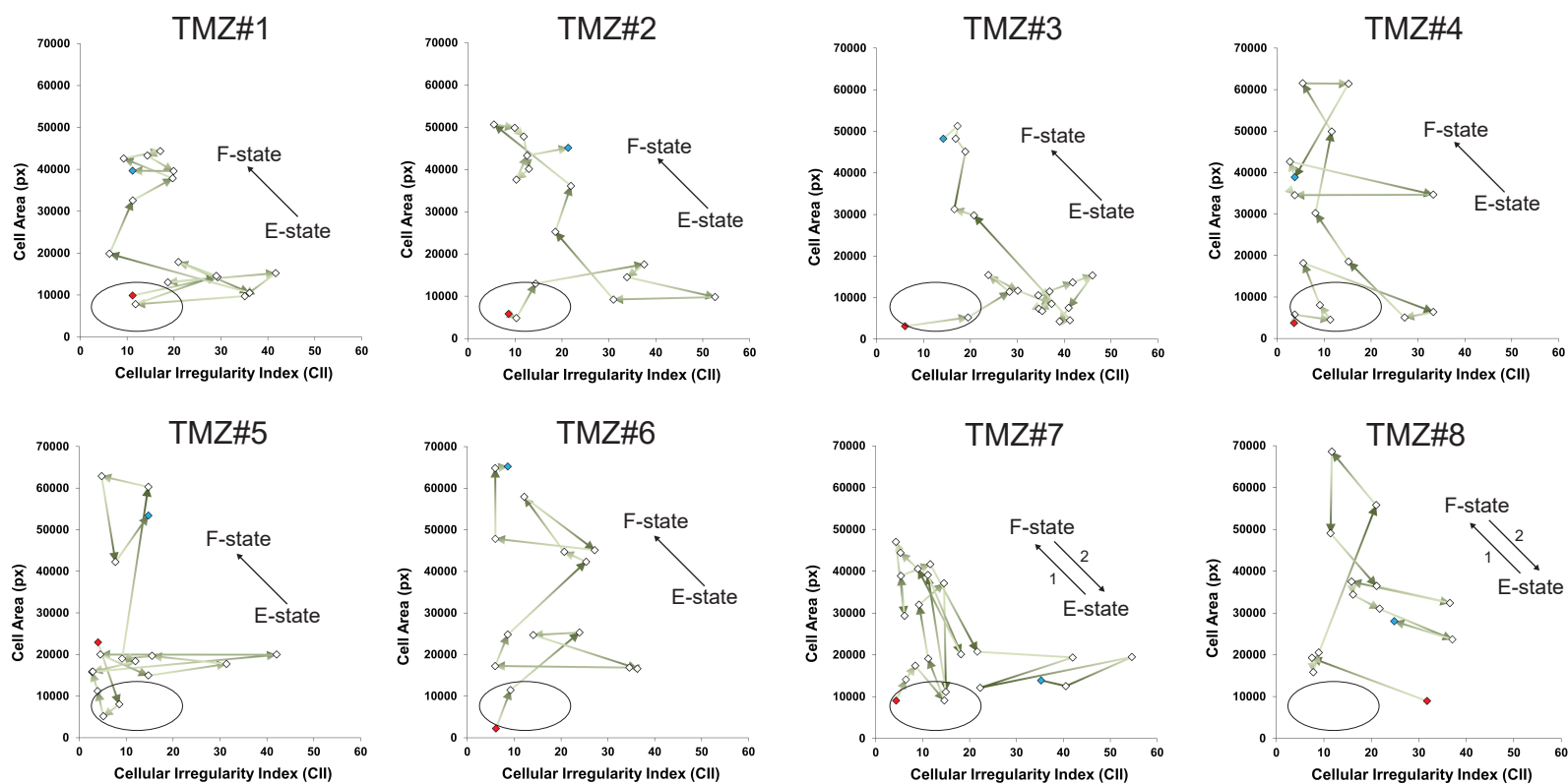

Supplement: Supplementary file 3 — Figure S3: TrackingCellMorph for control and TMZ conditions. Individual control and TMZ‐treated cells were tracked and segmented over time. (A) Control cells. (B) TMZ‐treated cells. [file ACEL-25-e70477-s006.pdf]

A

U87-FUCCI control cells

Brightfield

Fluorescence

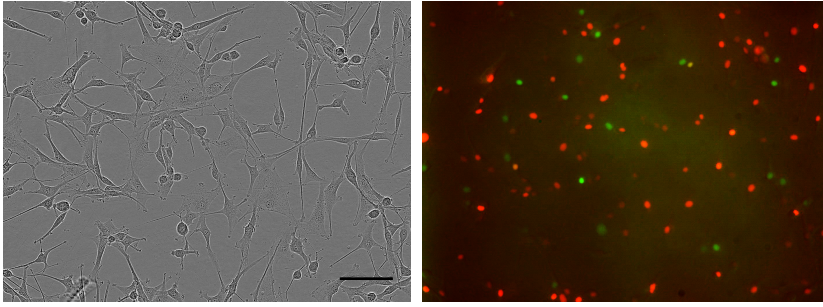

B

G1: 87%  
G2/M: 7%

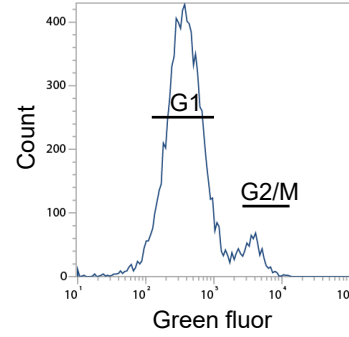

G1: 80%  
G2/M: 11%

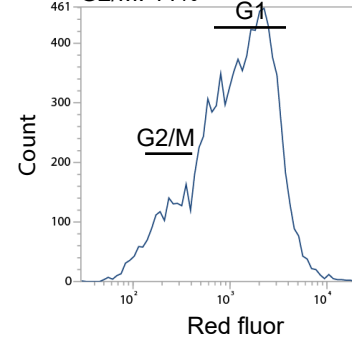

C

Control

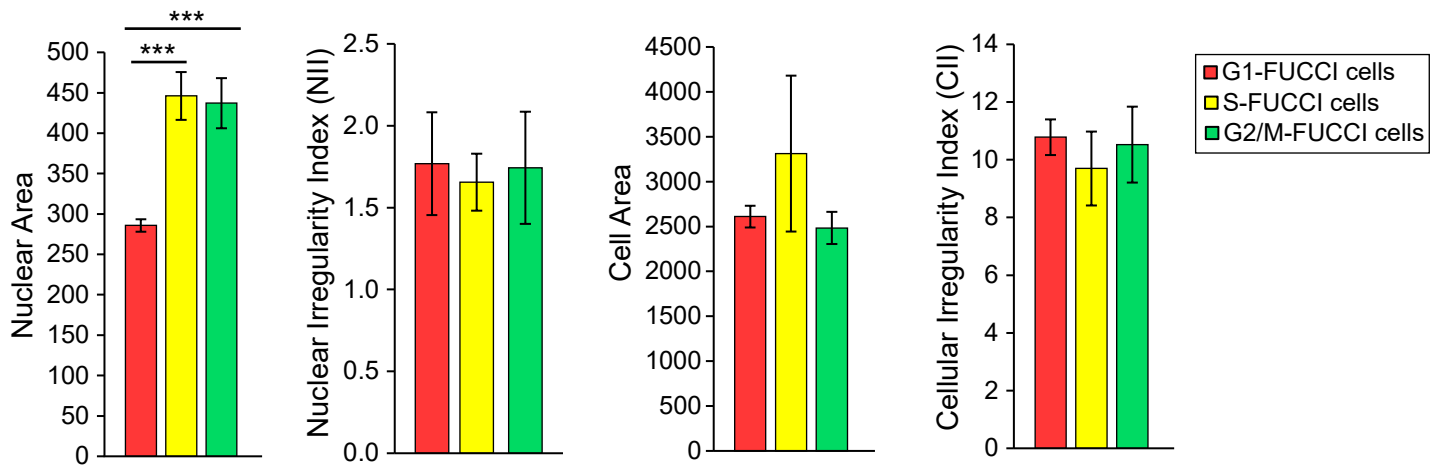

D

TMZ

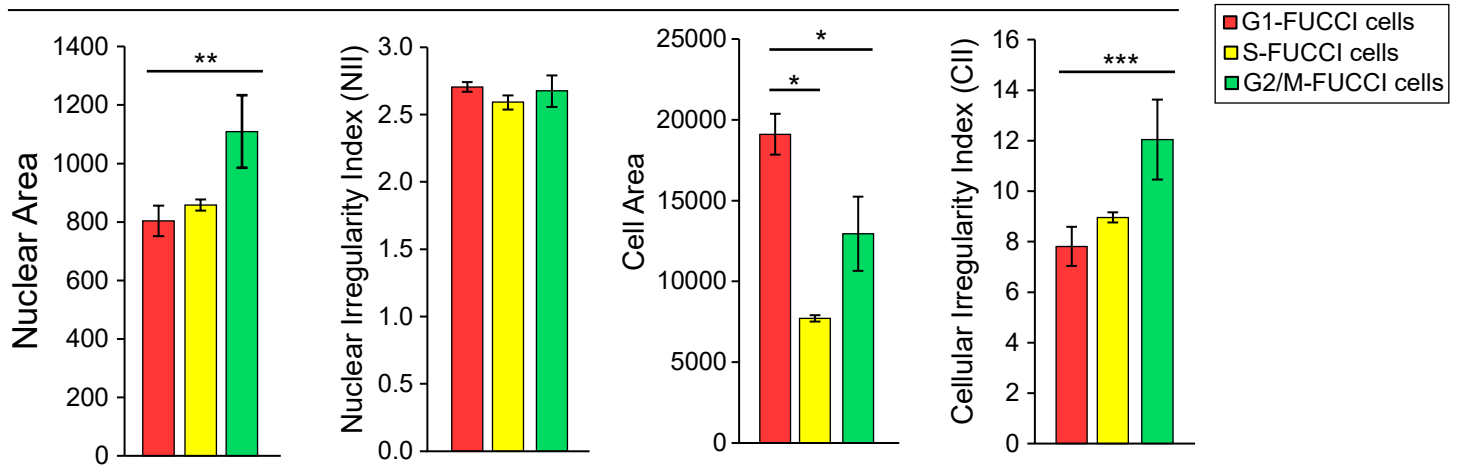

Supplement: Supplementary file 4 — Figure S4: Cell cycle distribution, cellular and nuclear morphometric features of U‐87‐FUCCI cells. (A) Representative images of control cells. (B) Flow cytometry of U‐87‐FUCCI cells of control condition. Left—cell cycle distribution considering green fluorescence, where the peak on the left (i.e., negative cells) represents the G1 phase. Right—cell cycle distribution considering red fluorescence, where the peak on the left (i.e., negative cells) represents the G2/M phase. (C and D) Nuclear and cellular morphometric features of control and TMZ‐treated cells, respectively, according to FUCCI patterns. *p < 0.05, **p < 0.01, ***p < 0.001. [file ACEL-25-e70477-s009.pdf]

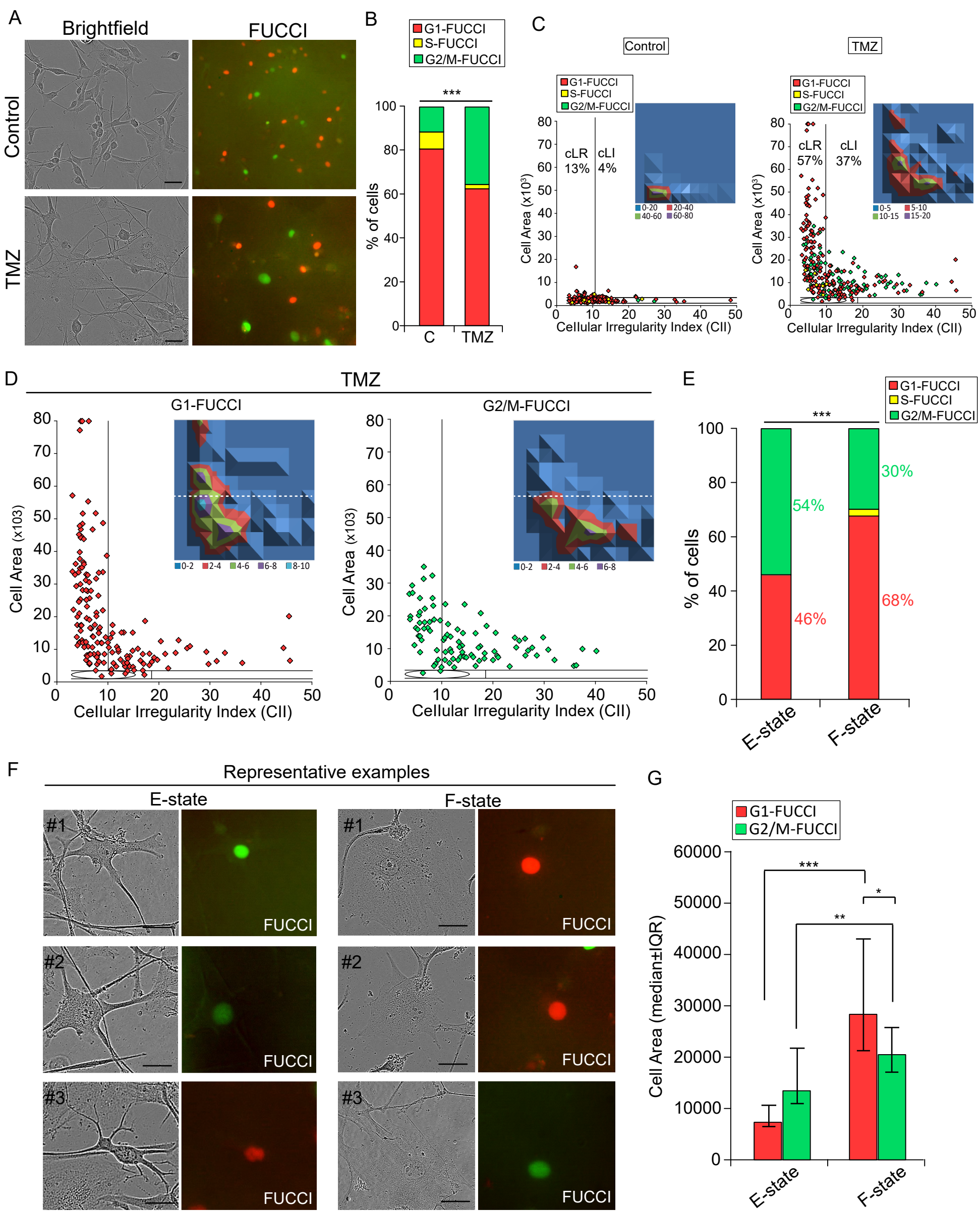

Supplement: Supplementary file 5 — Figure S5: Cell cycle status in E‐state and F‐state cells. U‐87 cells stably expressing the FUCCI reporter were treated with TMZ 50 μM for 5 days, followed by growth in Drug‐Free Medium. Individual cells were analyzed for cell cycle phase, NMA, and CellMorph in an integrated manner, 5 days after replating in DFM. (A) Representative images of control (top) and TMZ‐treated (bottom) U‐87‐FUCCI cells. (B) Cell cycle distribution for control and TMZ‐treated cells. ***p < 0.001 (chi‐squared test). (C) fucciCellMorph multiphenotypic analysis of control and TMZ‐treated cells. Red, yellow, and green markers represent cells in G1, S, and G2/M phases, respectively. The percentages of cells in cLR (F‐state cells) and cLI (E‐state cells) quadrants are also shown. Inserts represent density plots. (D) Specific analysis of G1 and G2 cells in the fucciCellMorph graph. The percentages of cells in cLR (F‐state cells) and cLI (E‐state cells), as well as density plots for each condition, are also shown. (E) Percentage of G1, S, and G2 cells considering E‐state (cLR) and F‐state (cLI) cells. ***p < 0.001 (chi‐squared test). (F) Representative E‐state and F‐state cells. (G) Cell area for G1 or G2/M cells considering E‐state (cLR) and F‐state (cLI) cells. *p < 0.01, **p < 0.01, ***p < 0.001 (Mann–Whitney). [file ACEL-25-e70477-s003.pdf]

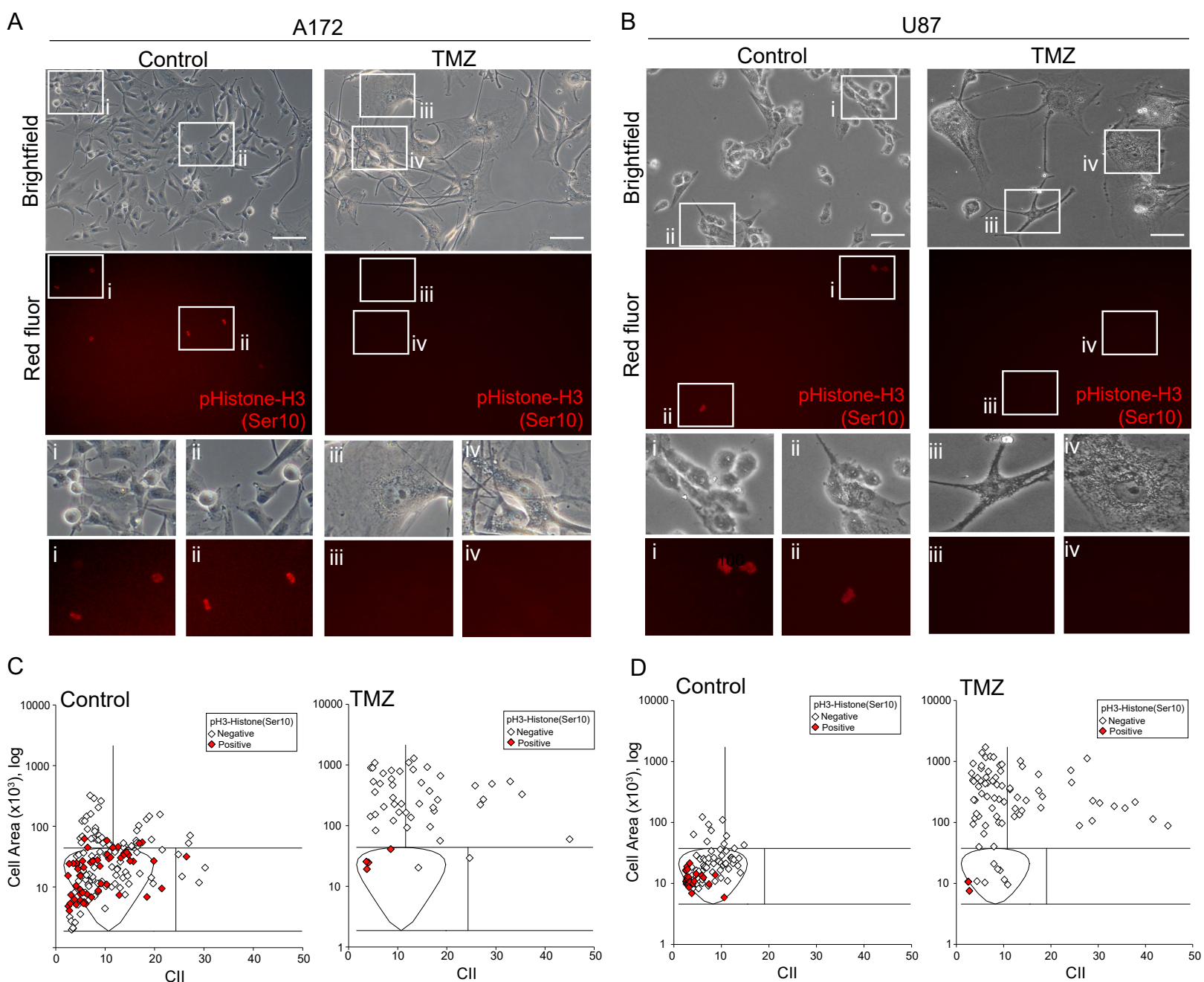

Supplement: Supplementary file 6 — Figure S6: TMZ‐treated enlarged A172 and U87 cells are negative for phospho‐HistoneH3(Ser10). A172 and U87 cells were treated with TMZ 50 μM for 5 days, followed by regrowth in Drug‐Free Medium. After 5 days, immunocytochemistry for phospho‐HistoneH3(Ser10) was performed. (A and B) Representative images of control and TMZ‐treated (A) A172 and (B) U87 cells. (C and D) CellMorph of control (left) and TMZ‐treated cells (right) for (C) A172 and (D) U87 cells. Phospho‐HistoneH3(Ser10)‐positive cells are shown in red. [file ACEL-25-e70477-s004.pdf]

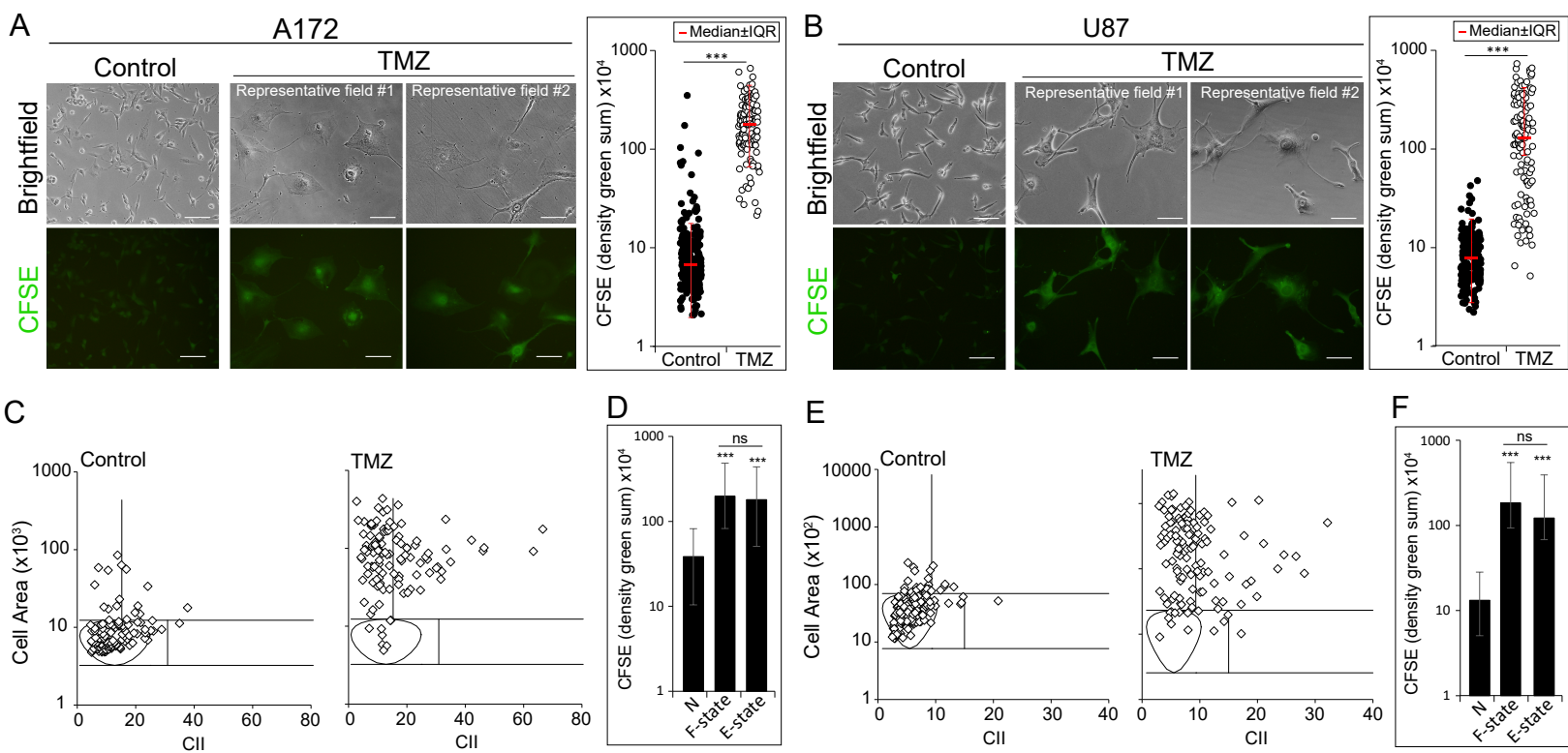

Supplement: Supplementary file 7 — Figure S7: CFSE fluorescence is higher in TMZ‐induced enlarged cells. A172 and U87 cells were treated with TMZ 50 μM for 5 days, followed by regrowth in Drug‐Free Medium. After 5 days, cells were incubated with CFSE. After 2 days, images were acquired, followed by cellular segmentation to extract information about cell size and shape, as well as the green fluorescence (density sum). (A and B) Left—Representative images (brightfield and green fluorescence) of control and TMZ‐treated conditions. Right—green fluorescence (density sum) for control and TMZ‐treated cells; data are shown as median ± IQR (interquartile range). (C) CFSE‐CellMorph graph for control and TMZ‐treated conditions in A172 cells. (D) Green fluorescence (density sum) for Normal (N), F‐state and E‐state cells in TMZ‐treated A172 cells; data are shown as median ± IQR (interquartile range). (E) CFSE‐CellMorph graph for control and TMZ‐treated conditions in U87 cells. (F) Green fluorescence (density sum) for Normal (N), F‐state and E‐state cells in TMZ‐treated U87 cells; data are shown as median ± IQR (interquartile range). [file ACEL-25-e70477-s008.pdf]

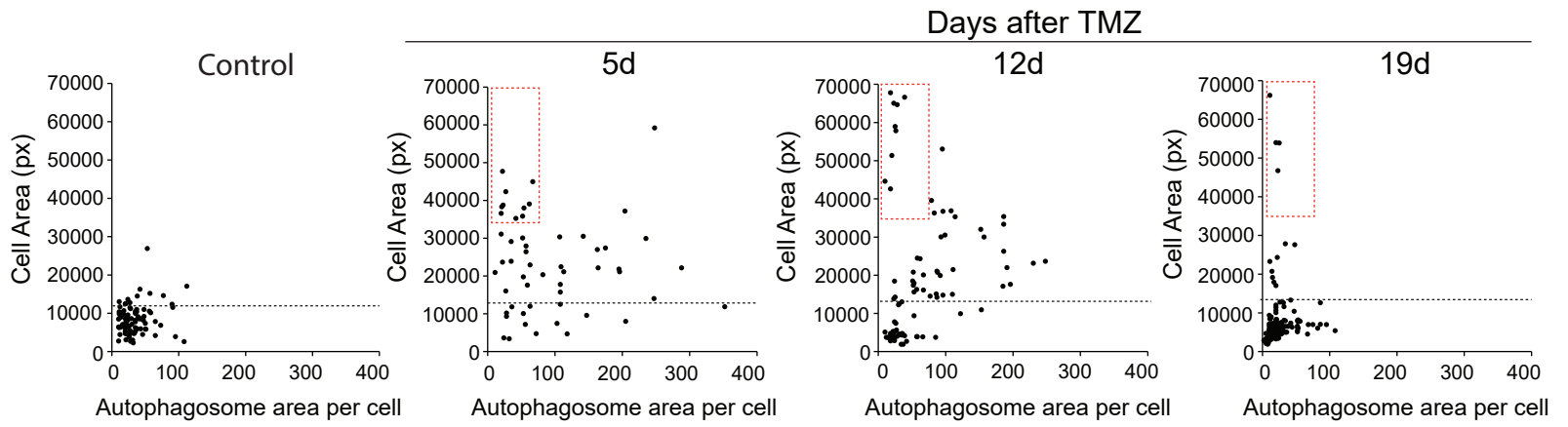

Supplement: Supplementary file 8 — Figure S8: Autophagy levels are increased during the transition to increased cell size. Autophagosomes area per cell versus cell area. Dashed black line: threshold separating normal and large cells. Red dashed line box—very large cells (i.e., cells with area higher than the average + 6SD, as shown in Figure S1B). [file ACEL-25-e70477-s002.pdf]

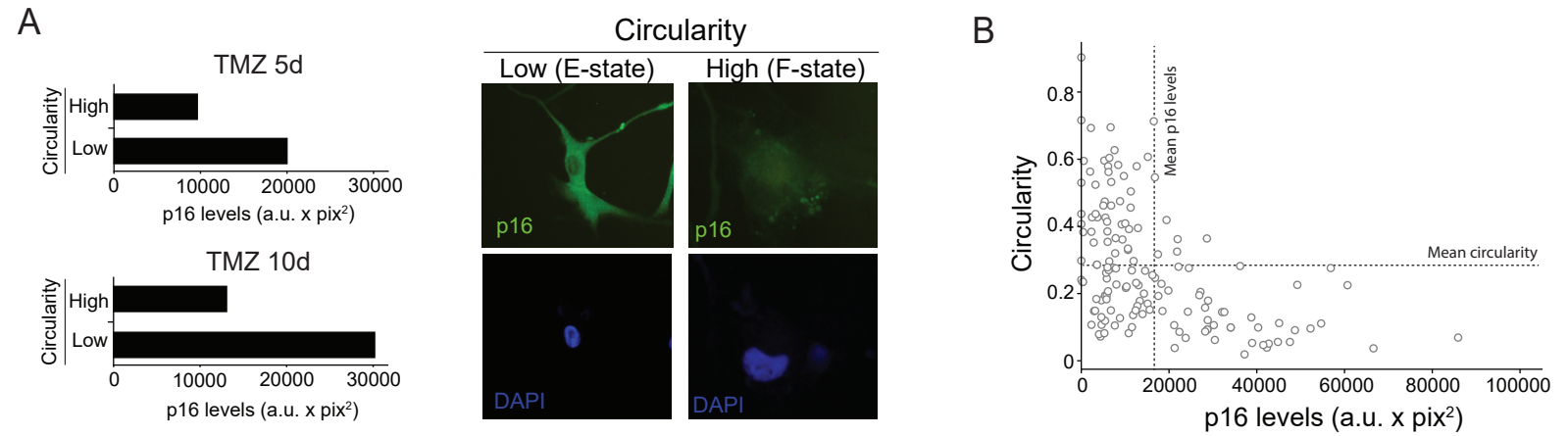

Supplement: Supplementary file 9 — Figure S9: Enlarged cells living in F‐state or E‐state differentially increase levels p16. (A) Levels of p16 according to the circularity. Left—the cells were separated according to the circularity into low and high, and then p16 levels were calculated in each group. Right—representative images. (B) p16 levels versus circularity scatterplot. [file ACEL-25-e70477-s005.pdf]

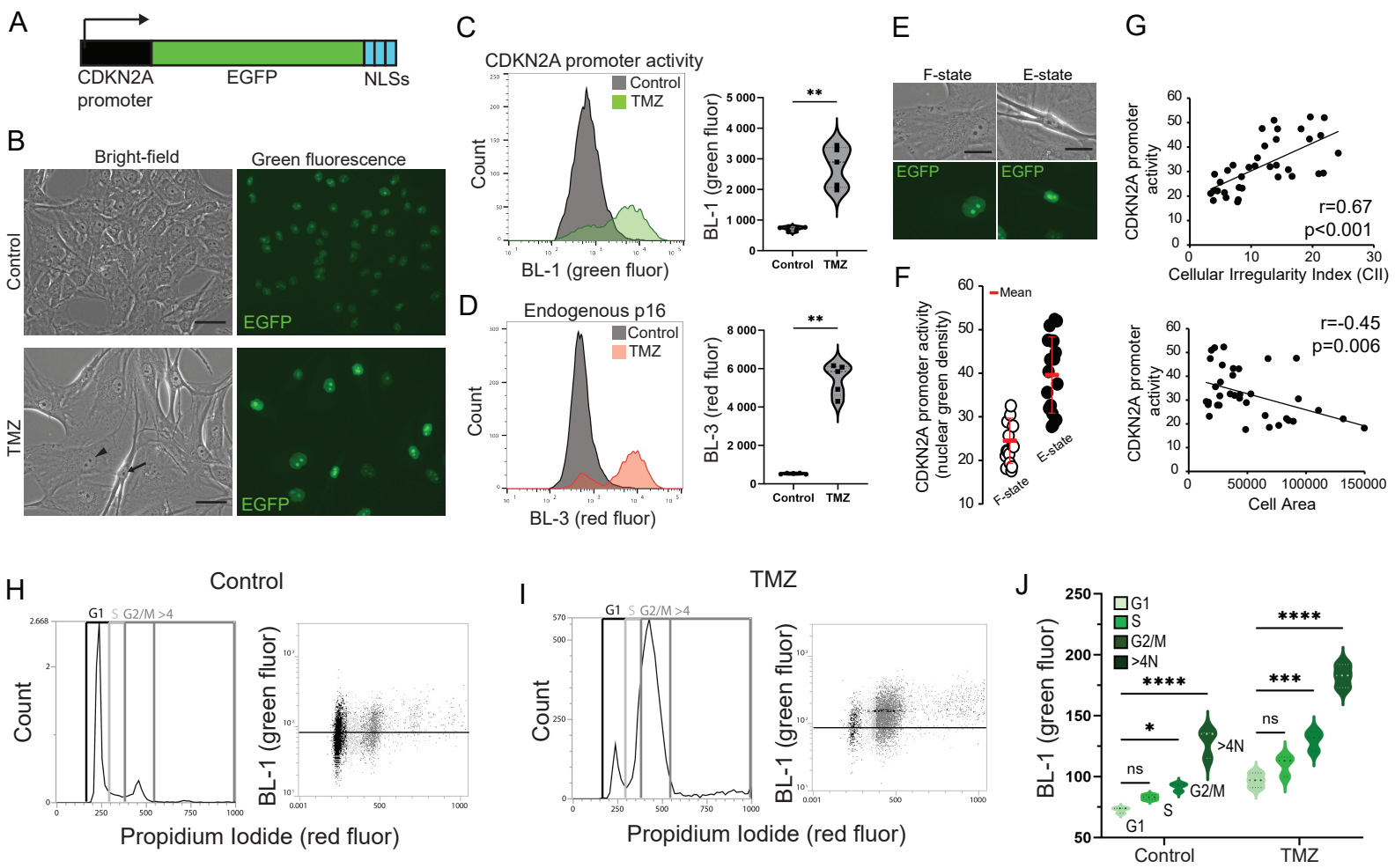

Supplement: Supplementary file 10 — Figure S10: Relationship between CDKN2A promoter activity and morphometry of A172 cells treated with TMZ. A172 cells were transduced with lentivirus to stably express the CDKN2A promoter controlling the expression of EGFP (named FluoSen reporter). EGFP was tagged with three nuclear localization sequences (NLS). (A) Simplified diagram of the fluorescent reporter. A172‐FluoSen cells were treated with TMZ 50 μM for 5 days, followed by reseeding in Drug‐Free Medium. (B) Representative images of control and TMZ‐treated A172‐FluoSen cells. (C) Flow cytometry analysis to measure green fluorescence levels in control and TMZ‐treated cells. (D) Flow cytometry analysis of endogenous p16 levels, measured in the BL3 (red) channel. (E) Representative images of E‐state (top) and F‐state (bottom) cells with nuclear fluorescence representing the activity of the CDKN2A promoter. (F) Differential CDKN2A promoter activity in E‐state and F‐state cells. (G) Top—correlation between CDKN2A promoter activity and Cellular Irregularity Index (CII). Bottom—correlation between CDKN2A promoter activity and Cell Area. (H and I) Cell cycle analysis of A172‐FluoSen cells using propidium iodide (PI) staining. Both histograms for PI (left) and scatterplot for PI versus green fluorescence (right) are shown. (J) Green fluorescence levels in cells in different phases of the cell cycle. [file ACEL-25-e70477-s001.pdf]

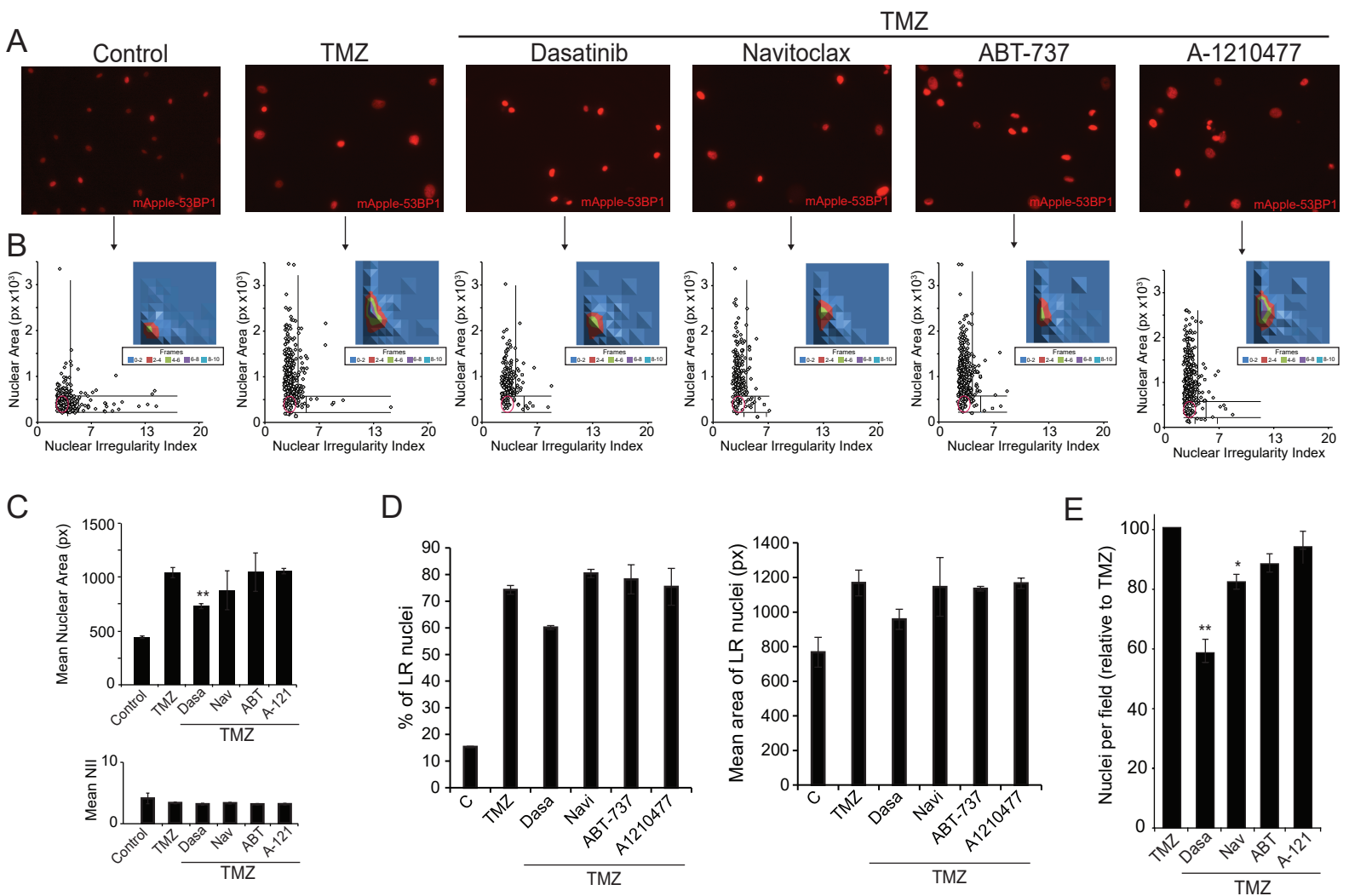

Supplement: Supplementary file 11 — Figure S11: Senolitycs have differential effects in TMZ‐treated glioblastoma cells. Cells were treated with senolytics on Day 10 after TMZ, for 24 h. (A) Representative nuclei of control cells and cells treated with TMZ or TMZ plus senolytics. (B) NMA scatterplot. Inserts show the NMA density plot. (C) Top—mean Nuclear Area; bottom—mean NII. (D) Left—the percentage of nLR; Right—mean area of LR nuclei. (E) Nuclei per field (relative to TMZ, considered 100%). [file ACEL-25-e70477-s010.pdf]

A

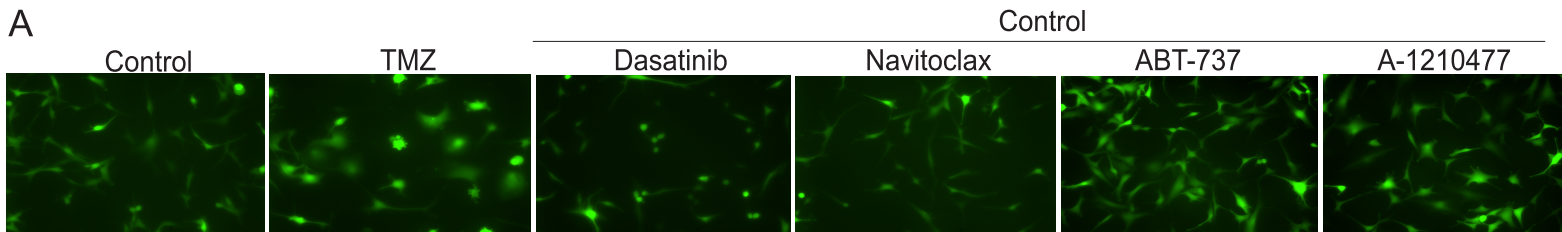

B

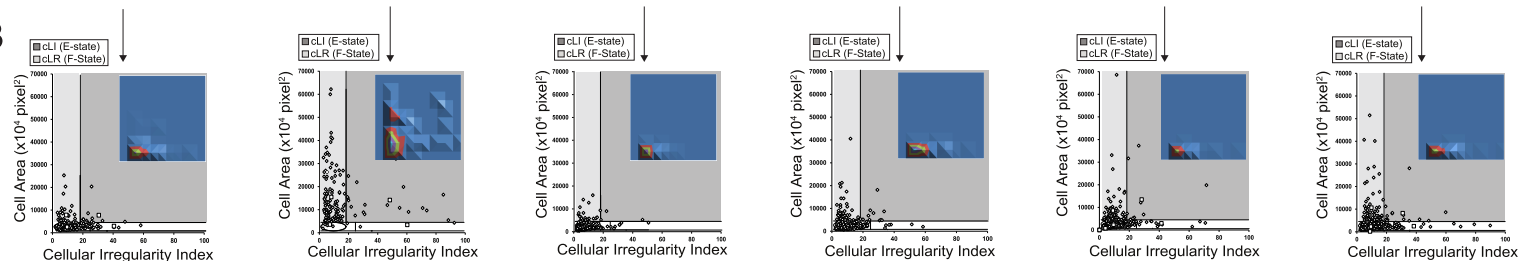

C

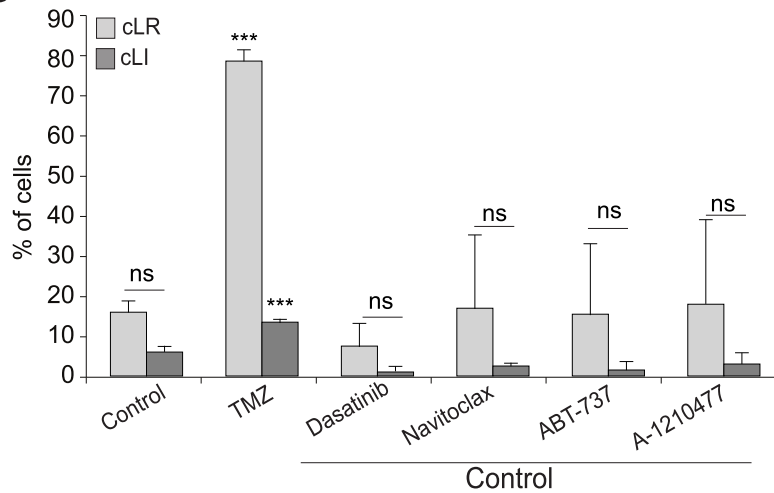

D

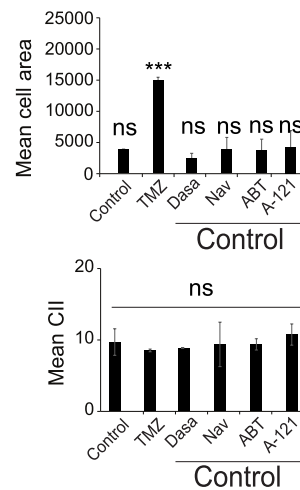

E

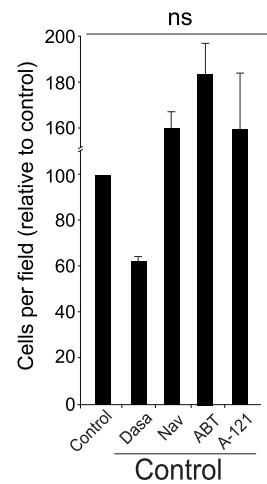

Supplement: Supplementary file 12 — Figure S12: Senolytics effects over non‐treated glioblastoma cells. Cells were treated with senolytics on Day 10, for 24 h. (A) Representative control cells and cells treated with senolytics. (B) CellMorph scatterplot. Inserts show CellMorph density plot. cLI, Large Irregular cells (E‐state); cLR, Large Regular cells. (C) Percentage of cells at cLR and cLI. (D) Mean cell area and CII. (E) Number of cells per field relative to Control (considered 100%). [file ACEL-25-e70477-s007.pdf]
